# Anti-Müllerian Hormone Type II Receptor Expression in Endometrial Cancer Tissue

**DOI:** 10.3390/cells9102312

**Published:** 2020-10-17

**Authors:** Marek Gowkielewicz, Aleksandra Lipka, Marta Majewska, Aleksandra Piotrowska, Marta Szadurska-Noga, Jacek J. Nowakowski, Marta Wiszpolska, Piotr Dzięgiel, Tomasz Wasniewski, Mariusz Krzysztof Majewski, Marcin Jozwik

**Affiliations:** 1 Department of Gynecology and Obstetrics, School of Medicine, Collegium Medicum, University of Warmia and Mazury in Olsztyn, 10-082 Olsztyn, Poland; aleksandra.lipka@uwm.edu.pl (A.L.); tomasz.wasniewski@uwm.edu.pl (T.W.); marcin.jozwik@uwm.edu.pl (M.J.); 2 Department of Human Physiology and Pathophysiology, School of Medicine, Collegium Medicum, University of Warmia and Mazury in Olsztyn, 10-082 Olsztyn, Poland; marta.majewska@uwm.edu.pl (M.M.); marta.wiszpolska@uwm.edu.pl (M.W.); mariusz.majewski@uwm.edu.pl (M.K.M.); 3 Division of Histology and Embryology, Department of Human Morphology and Embryology, Wroclaw Medical University, 50-368Wroclaw, Poland; aleksandra.piotrowska@umed.wroc.pl (A.P.); piotr.dziegiel@umed.wroc.pl (P.D.); 4 Department of Pathomorphology, School of Medicine, Collegium Medicum, University of Warmia and Mazury in Olsztyn, 10-561 Olsztyn, Poland; szadurskamarta@gmail.com; 5 Department of Ecology & Environmental Protection, University of Warmia and Mazury in Olsztyn, 10-727 Olsztyn, Poland; jacek.nowakowski@uwm.edu.pl; 6 Department of Physiotherapy, Wroclaw University School of Physical Education, 51-612 Wroclaw, Poland

**Keywords:** endometrial cancer, AMHRII, AMH, expression, tissue microarray

## Abstract

Anti-Müllerian hormone (AMH) is responsible for the Müllerian ducts’ regression in male fetuses. In cells of cancers with AMH receptors (AMHRII), AMH induces cell cycle arrest or apoptosis. As AMH occurs naturally and does not exhibit significant side effects while reducing neoplastic cell colonies, it can be considered as a potential therapeutic agent for cancer treatment. The purpose of this study was to assess the AMHRII expression in endometrial cancer (EC) in correlation to various demographic data and clinical conditions. Immunohistochemical analysis was used to assess AMHRII expression in EC tissue samples retrieved from 230 women with pre-cancerous state of endometrium (PCS) and EC. AMHRII was detected in 100% of samples. No statistical difference was observed for AMHRII expression depending on the histopathological type of EC, cancer staging, body mass index, and age, as well as the number of years of menstruation, births and miscarriages, and average and total breastfeeding time. Diabetes mellitus type 2 is the only factor that has an impact on AMHRII expression in EC tissue. Thus, this study supports the idea of theoretical use of AMH in EC treatment because all histopathological types of EC at all stages of advancement present receptors for AMH.

## 1. Introduction

During embryo development, the Müllerian ducts in females differentiate into the fallopian tubes, uterus, cervix, proximal vagina, and surface epithelium of the ovaries. In male fetuses, anti-Müllerian hormone (AMH) induces regression of the precursors to those structures [1]. The *AMH* gene, located in chromosome 19p13.3, contains five exons and encodes a 140-kDa dimeric glycoprotein, which belongs to the transforming growth factor β (TGF-β) superfamily [2]. As other factors in the TGF-β superfamily, AMH binds to the serine-threonine kinase receptor complex. AMH signal transduction requires interaction of two similar but distinct receptors: AMH receptor type I (AMHRI) and AMH receptor type II (AMHRII). However, AMHRII is the primary receptor [3]. In short, AMH attaches directly to the unique AMHRII, which then binds type I receptor [4]. Such a complex activates the SMAD protein and the other signaling cascades, which triggers transcription factors to induce gene expression, apoptosis, and regression of the Müllerian ducts [5].

The *AMHRII* gene, localized on chromosome 12q13, encompasses 11 exons spread over 8 kb. The extracellular domain that binds the ligand is encoded by the first three exons, the fourth exon encodes the single transmembrane domain, and the last seven exons encode the intracellular serine/threonine kinase domain [5]. Due to the orientation of the N-terminus to the outside and the presence of a signal sequence (responsible for translocation to the endoplasmic reticulum), AMHRII is classified as a type I membrane protein [6].

Beside an important role in fetal sexual differentiation AMH may inhibit the growth of tumors that mainly originate from the Müllerian ducts and express AMHRII at a high frequency [3], including ovarian [7], cervical [8], endometrial [9], breast [10], and prostate cancer [11], and even ocular melanoma [12]. Additionally, AMH may improve the effectiveness of classical chemotherapeutics, reducing needed doses and decreasing its toxicity [13]. Endometrial cancer (EC) is the most common gynecologic malignancy [14]. EC includes a variety of tumor types with diverse microscopic features, genetic background, and prognoses. Additionally, hormonal influence interacts with genetic alterations in the pathogenesis and growth regulatory pathways of at least some types of EC [13].

Low AMHRII expression is present in healthy endometrium (both phases) of premenopausal women and also atrophic endometrium, while in endometrial hyperplasia and endometrial cancer, elevated AMHRII protein expression occurs [15]. However, this research concerned a relatively small group of samples and did not assess the AMHRII presence in rare but more aggressive types of EC from the second group according to Bokhman’s division: clear cell, mixed or serous, biologically similar to ovarian cancers. So far, researchers’ efforts have focused mainly on the use of AMHRII presence in the treatment of serous ovarian cancer as one of the most aggressive types of cancer. Anti-AMHRII-radiolabeled antibodies could be proposed to ovarian cancer patients as an alternative adjuvant treatment after cytoreductive surgery, thus it seems to be a realistic theranostic option for the clinic [16]. AMHRII expression should be further investigated as a potential therapeutic target in other gynecologic cancer tissues [17], especially including the aggressive EC type. So far, among new targeted therapy agents for EC, only pembrolizumab has been approved [18]. There is a need for new solutions in oncology to manage advanced, recurrent, and metastatic endometrial cancers [18,19]. Therefore, the aim of this study was to investigate AMHRII expression in different types of endometrial cancer. Additionally, the obtained results were correlated with medical data concerning comorbidities and patients’ features.

## 2. Materials and Methods

### 2.1. Patients

The study was performed on archived paraffin blocks of EC obtained from 230 patients who underwent surgical removal of precancerous or cancerous endometrial lesions (hysterectomy with bilateral salpingo-oophorectomy in most cases accompanied by lymphadenectomy, as well as peritoneal washing at the Clinical Ward of Gynecology, Obstetrics and Oncological Gynecology at the Regional Specialist Hospital in Olsztyn, Poland. The study protocol was approved by the Bioethics Committee of the Warmia-Mazury Medical Chamber (OIL. 164/15/Bioet; 2 April 2015) in Olsztyn, Poland. Case history reviews were collected for all patients in order to record demographic details and tumor characteristics, including comorbidities and survival outcomes. Biopsy samples from the affected area were processed as previously described [19]. Briefly, samples were preserved in 4% buffered formaldehyde in the form of a ready-to-use phosphate-buffered solution (Chempur, Poland) immediately after surgical removal of tissue. The volume of fixative to tissue ratio was at least 10:1. All samples received a minimum of 6 h and maximum of 12 h of fixation at room temperature (22–25 °C).

### 2.2. Tissue Microarrays (TMAs)

All paraffin blocks were cut into 6-μm-thick sections that were hematoxylin and eosin (H&E) stained and evaluated by an experienced pathologist. Further, the prepared slides were scanned using the Pannoramic MIDI II (3DHistech; Budapest, Hungary) histological scanner. PannoramicViewer (3DHistech) software was used to manually select three representative areas of a surface of 1.5 mm^2^, from regions of EC previously indicated by the pathologist. Three representative cores of 1.5-mm diameter were taken from each archival sample of an EC tumor and embedded in paraffin to create tissue microarrays (TMAs) using TMA Grand Master (3DHistech) in line with the manufacturer’s instructions.

### 2.3. Immunohistochemistry (IHC)

Slides from TMAs (4 μm thick) were used for IHC reactions, which were performed using DakoAutostainer Link48 (Dako; Glostrup, Denmark). Boiling (97 °C) for 20 min in EnVision FLEX Target Retrieval Solution (pH 9) at the PTLink platform (Dako; Glostrup, Denmark) was used to deparaffinize, rehydrate the sections, and unmask the antigens. Further, the activity of endogenous peroxidase was blocked by incubating slides for 5 min with Envision Flex Peroxidase-Blocking Reagent (Dako, Glostrup, Denmark). To detect AMHRII in the EC samples, slides were incubated for 20 min with primary antibodies against AMHRII (ab197148; 1:1600, Abcam). Then, they were incubated with EnVision FLEX/HRP (20 min), and the reaction was visualized due to incubation (10 min) in freshly prepared 3,3′-diaminobenzidine (DAB). Additionally, slides were counterstained (5 min) with EnVision FLEX Hematoxylin (Dako; Glostrup, Denmark). Finally, they were dehydrated in ethanol (70%, 96%, absolute) and xylene, then mounted with Dako Mounting Medium (Dako; Glostrup, Denmark). A positive control tissue was a human prostate. All immunohistochemical reactions were evaluated (BX-41 light microscope, Olympus, Tokyo, Japan) by two pathologists using the Remmele and Stegner score [20].

### 2.4. Other Definitions

According to the World Health Organization, the body mass index (BMI) is a person′s weight (in kilograms) divided by the square of their height (in meters). Someone with a BMI equal to or more than 25 is overweight. A BMI of 30 or more means that an individual is obese.

According to the British guidelines (2015) from the National Collaborating Centre for Women’s and Children’s Health, the perimenopausal period is the time of irregular menstruations and vasomotor symptoms. The menopausal period means that a woman has not had menstruation for at least 12 months and she does not use hormonal contraception [21].

### 2.5. Statistical Analysis

The expression of AMHRII was measured with the semi-quantitative rank scale ISR in three samples retrieved from three places of cancer tissue from each patient. The mean value of AMHRII expression for each patient was computed, and the mean values were used in the whole analysis. To choose an appropriate approach in further statistical analysis, all samples were tested for compliance with the normal distribution by the Shapiro–Wilk test. In some samples, the distributions were not consistent with the theoretical normal distribution (Supplementary Materials Figures S1 and S2), and also AMHRII expression was measured with the rank scale type, which did not correspond to the density of the distribution typical for the theoretical normal distribution, and which was the premise for nonparametric testing. Because significant multivariate models of the relationship between AMHRII expression and variability of independent variables and interactions between them were not found, only the results of univariate tests were presented. Differences in AMHRII expression between the type of cancer, cancer stages according to FIGO, and hormonal status of women were tested with the Kruskal–Wallis test. Results of the comparisons between individual groups were based on the post hoc nonparametric multiple comparison tests.

The comparison of AMHRII expression between two examined groups of patients, e.g., presence of diabetes (type 2), presence of hypertension, and use of hormonal replacement therapy (HRT), was performed with the Mann–Whitney U test. The relationship between AMHRII expression and the value of metric traits, e.g., age, years of menstrual activity, number of births, miscarriages, time of breastfeeding, and BMI, was tested using Spearman’s rank correlation coefficient. The *p*-value < 0.05 was defined as statistically significant. Statistical analysis was performed using Statistica 13.0 software (StatSoft, TIBCO Software Inc., Palo Alto, CA, USA, 2014).

## 3. Results

**Figure 1:**
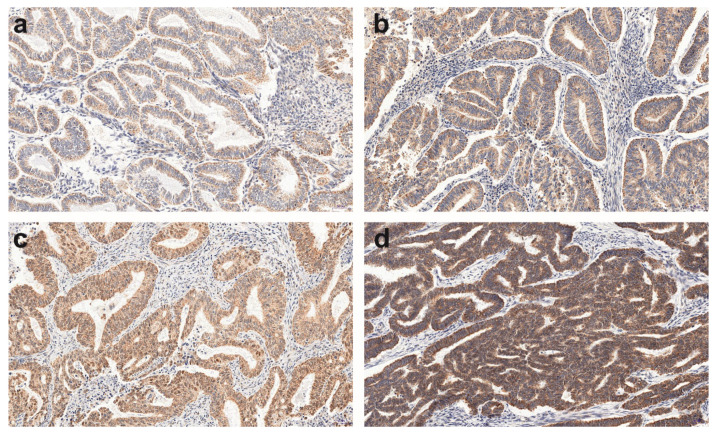
A representative example (20 × magnification) of the weak (**a**), moderate (**b, c**), and strong (**d**) AMHRII immunohistochemistry (IHC) reaction within endometrioid adenocarcinoma G1 (**a**) and G2 (**b**–**d**) grade.

Among 230 TMAs specimens, 230 showed a positive AMHRII reaction (Figure 1) and were further analyzed. The detected AMHRII expression and its mean values of the immunoreactive score of Remmele and Stegner [20] are presented in Table 1.

**Figure 2:**
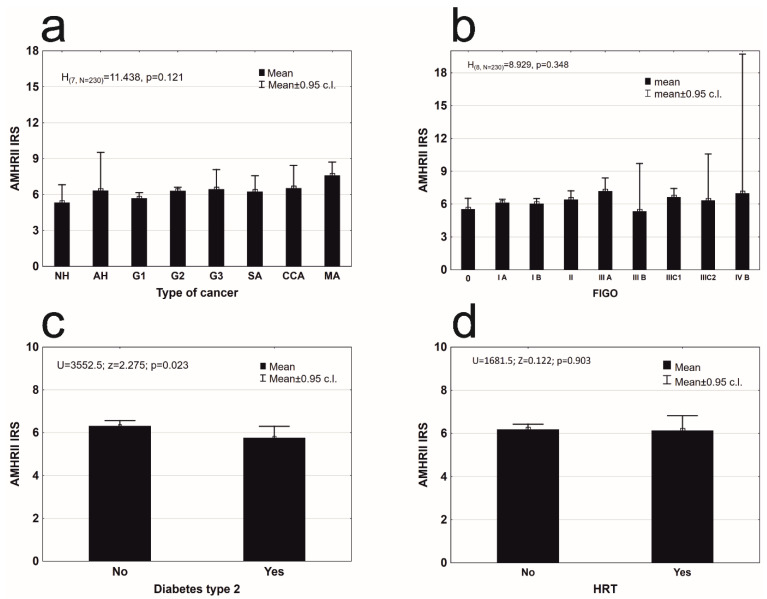
Mean AMHRII expression in: (**a**) different types of endometrial lesion (description of histopathological groups: NH, AH, G1, G2, G3, SA, CCA, MA in Table 1) (Kruskal–Wallis test, H_(7, N = 230)_ = 11.438, *p* = 0.121); (**b**) in different clinical stages of endometrial cancer according to FIGO (Kruskal–Wallis test, H_(8, N = 230)_ = 8.928, *p* = 0.348); (**c**) group of patients without and with diabetes mellitus type 2 (Mann–Whitney U test, AMHRII: Z = 2.275, *p* = 0.023); (**d**) group of patients that used hormone replacement therapy (Wald–Wolfowitz runs test, Z = 0.122, *p* = 0.923).

All specimens were divided into eight groups, based on their histopathological type: nonatypical hyperplasia (8), atypical hyperplasia (4), endometrioid adenocarcinoma G1 (49), G2 (146), G3 (6), serous adenocarcinoma (8), clear cell adenocarcinoma (4), and mixed adenocarcinoma (5). AMHRII was found in tissues of all types of cancers at a similar level of expression (Figure 2a).

**Figure 3:**
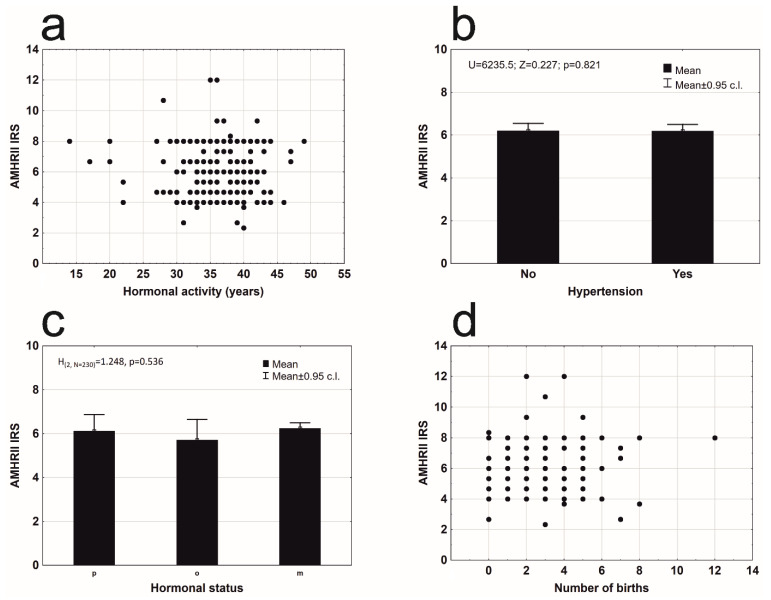
Mean AMHRII expression: (**a**) due to years of hormonal activity (r_s_ = 0.053, n = 230, *p* = 0.426); (**b**) in a group of patients without and with arterial hypertension (Mann–Whitney U test: Z = 0.227, *p* = 0.821); (**c**) due to hormonal status: premenopausal (*p*), perimenopausal (o), and postmenopausal (m) women (Kruskal–Wallis test: H_(2, N = 230)_ = 1.248, *p* = 0.536); (**d**) depending on the number of birth (r_s_ = 0.033, n = 230, *p* = 0.624).

An analysis of other factors that may have influenced AMHRII expression was conducted. AMHRII expression was present in all clinical stages of cancer according to FIGO (Figure 2b). A significant difference was not found between the degree of AMHRII expression and the degree of clinical staging of cancer (Figure 2b). Patients with diagnosed type 2 diabetes (53 cases) have significantly lower expression of AMHRII than patients without diabetes type 2 (Figure 2c). Women who used hormone replacement therapy (HRT) had statistically non-different levels of expression of the AMHR2 receptor protein, from a group of women who did not use such a therapy Figure 2d). There were no statistically significant differences in AMHR2 expression depending on the number of years of hormonal activity (Figure 3a). Hypertension did not affect the level of AMHR2 expression (Figure 3b). There was also no statistically significant difference of AMHR2 expression depending on the hormonal status [21] of the studied women (Figure 3c) or the number of deliveries (Figure 3d).

**Figure 4:**
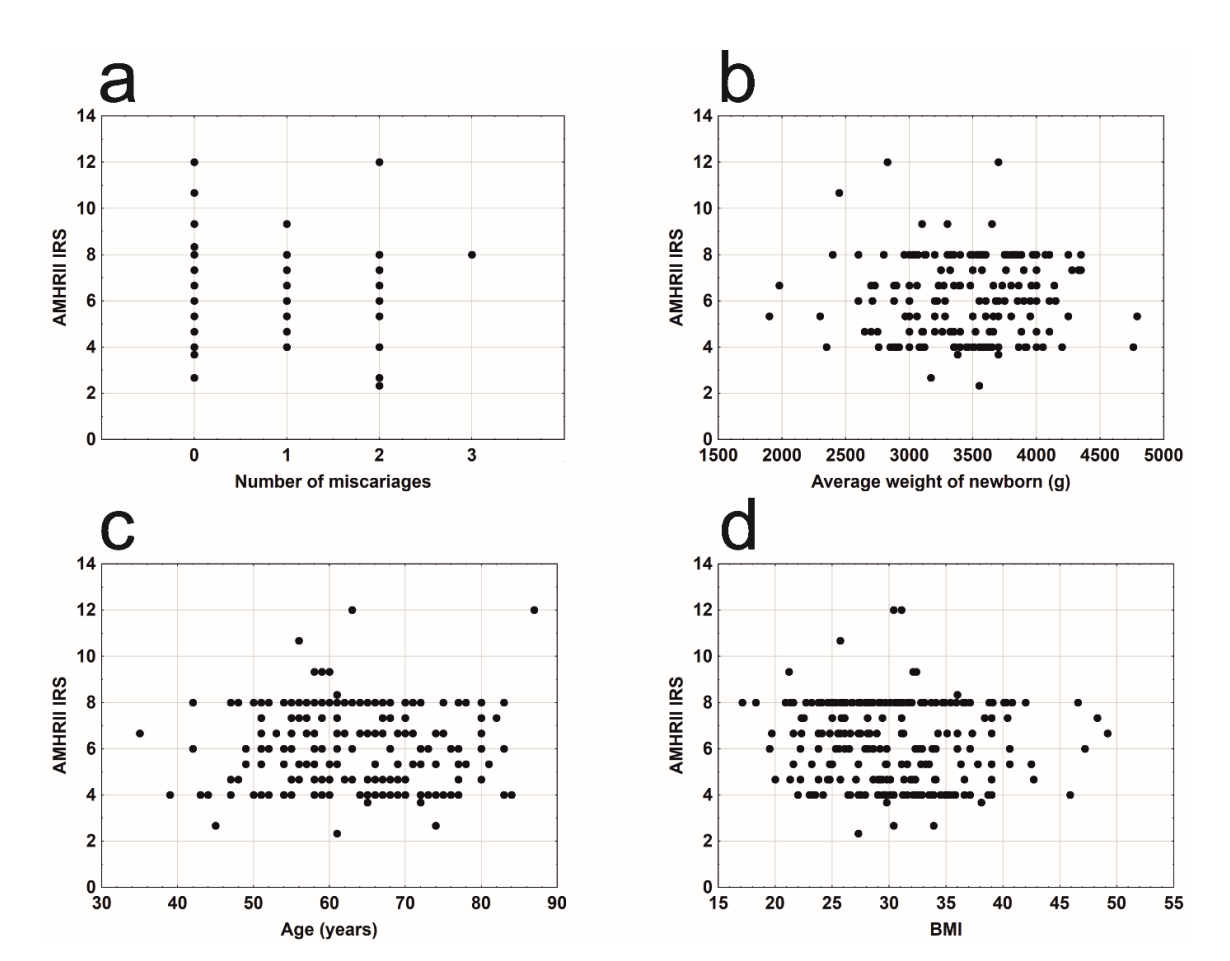
Mean AMHRII expression due to: (**a**) number of miscarriages (r_γ_ = −0.127, n = 230, *p* = 0.123); (**b**) average weight of newborn (s) (r_s_ = 0.102, n = 206, *p* = 0.146); (**c**) age of the patient (r_s_ = −0.002, n = 230, *p* = 0.980); (**d**) BMI (r_s_ = −0.088, n = 230, *p* = 0.183).

There were no significant relationships between AMHR2 expression and number of miscarriages (Figure 4a), average birth weight of children (Figure 4b), age of the examined women (Figure 4c), or BMI (Figure 4d).

**Figure 5:**
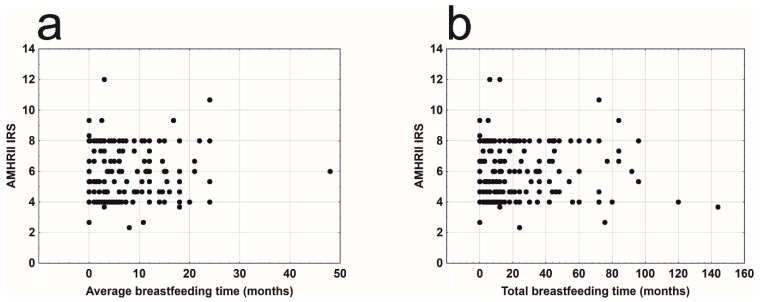
Mean AMHRII expression due to: (**a**) average breastfeeding time (r_s_ = −0.049, n = 227, *p* = 0.459); (**b**) total breastfeeding time (r_s_ = −0.030, n = 227, *p* = 0.650).

The average time of breastfeeding (Figure 5a) and the total time of breastfeeding (Figure 5b) also did not affect AMHRII expression in the analyzed patients.

## 4. Discussion

There is a paucity of data on conditions that modify the presence of AMH type II receptors (AMHRII) in EC tissues. The aim of the study was thus to identify whether the following factors may be involved: histopathological cancer type, stage of disease according to FIGO, BMI, parity, number of miscarriages, total and average time of breastfeeding, birth weight of the newborn, the length of hormonal activity of the ovaries, use of HRT, age at the disease diagnosis, hormonal status, concurrent diabetes, and hypertension.

Some of the factors mentioned above, exemplary of overweight and obese, have a well-documented effect on the risk of EC. The effect of being overweight and obese on the risk of EC is well documented [22]. Interesting data come from research into obesity and the overall mortality rate, which decreases in line with an increase of patients’ weight on the condition that they do not suffer from diabetes and are not nulliparous [23]. Among the analyzed patients in those studies, the average BMI was 30 to 45; however, the BMI value was not reflected in AMHRII expression. The issue of parity in the uterus EC cases is also unclear. The handbook knowledge presents nulliparity as a risk factor for EC, yet some studies on the topic indicate that multiparity does not prevent from EC development either [3]. Among the 230 patients in our study, above 80% gave birth to two or more children (almost half of them, i.e., 46.5%, gave birth to three or more children). Pregnancy is a period of prolonged exposure to progesterone, a hormone protecting the endometrium against cancer. However, as it is not correlated with AMHRII expression, there are probably other factors that counteract this beneficial effect of progesterone. Analyzing correlations between HRT and death caused by EC, it may be observed that hormone therapy reduces [24] or does not influence [23] the risk of death related to EC. The obtained results show that neither the length of breastfeeding time (both: total and average) nor the birth weight of newborns or their number have a significant effect on AMHRII expression. Late menarche is inversely proportional to the risk of EC while late menopause is directly proportional to the risk of EC [25]. However, analyses revealed that the period of estrogen activity has no effect on differences in AMHRII presence. Though, it was noticed that a period of hormonal activity longer than 40 years has a positive effect on the presence of AMH into endometrial cancer tissue and thus probably reduces the malignancy and spread of the cancer [19]. The above facts can describe the mechanisms controlling AMHRII expression in EC tissues as independent from sex hormone activity.

Confirmation of the presence of AMHRII in EC tissue may play an important role in modern approaches to EC staging and treatment. The Immuno-PET (positron emission tomography) technology, which employs radioactive labels and properties of monoclonal antibodies targeted at a particular antigen, is a tool enabling assessment of the quantity of metastases or neoplasm response to treatment [26]. Subjecting humans to radioactive zirconium-89 (^89^Zirconium)-labeled monoclonal antibodies has been medically validated. This is performed by applying nimotuzumab, which shows a strong affinity to the epidermal growth factor receptor (EGFR) [27], which is markedly often present in head and neck carcinomas [28]. Another agent that is applied is pertuzumab with an affinity to human epidermal growth factor receptor 2 (HER2), which occurs in HER2-positive breast cancer [29]. ^89^Zr-labeled antibodies against AMHRII were used in the detection of intraperitoneal xenograft from the cells of endometrial cancer (AN3CA line) in a mice model [16]. AMHRII expression was confirmed in, among others, prostate cancer, breast cancer, and ovary cancer [3,7,10,11]. It can be hypothesized that the application of anti-AMHRII antibodies conjugated with radioactive labels may be effective in primary staging or the assessment of relapses of the above diseases.

Radioimmunotherapy of intraperitoneal xenografts of EC cells with AMHRII (animal model), using AMHRII antibodies conjugated to radioactive isotopes of Lutetium (^177^Lu) and Bismuth (^213^Bi), provides positive clinical effects [16]. It is suggested that this modern theranostic type of therapy will be applied in the future in the treatment of intraperitoneal micrometastases of ovarian cancer, as well as other carcinomas [16]. We confirmed the presence of AMHRII in all stages of clinical advancement of EC according to FIGO classification. This provides a rationale to employ treatments based on AMHRII even in advanced stages of EC.

Even AMH alone has potential to be a safe anticancer agent [3,7,8,9,10,11,12,13]. Normal AMH concentrations in the serum of healthy women of childbearing age usually do not exceed 5 ng/mL [30]. In women with polycystic ovary syndrome (PCOS), this norm is exceeded a few to more than a dozen times [31]. Considerably higher AMH levels—from 1000 ng/mL to above 3000 ng/mL—are found in patients with granulosa cell tumors and sex cord tumors [30]. There are no reports confirming that increased serum AMH levels negatively impact the clinical condition of a patient without correlation with other factors. AMH, which is believed to be a potentially non-toxic substance with a beneficial therapeutic index, could be applied in adjuvant therapy alone, or as a carrier for other treatment agents. Then, it would limit their negative effect on tissues with AMHRII [3].

Binding to AMHRII, AMH acts via various proteins and signaling pathways. Cells of endometrial cysts of the ovary and gynecological cancers—cancers of the cervix, endometrium, and ovary—respond to the presence of AMH by inducing cell cycle arrest and apoptosis [7,8,9]. In endometrial cysts of the ovary, AMH increases the concentration of p21 protein, which is dependent on p53 protein and Rb factors, and p107 and p130 proteins. The concentrations of p16, p21, and E2F1 increase in ovarian cancer [9]. In cervical cancerous cells, concentrations of p16, E2F1, p107, and p130 increase as a result of applying AMH, while in endometrial cancer, these are concentrations of p107 and p130 [8,32].

In breast cancer and prostate cancer cell lines, AMH induces cell cycle arrest, activating a protein complex that regulates transcription. The complex is called the pathway of nuclear factor kappa-light-chain-enhancer of activated B cells (NFκB) [10,33]. Via NFκB, AMH induces IEX-1 (immediate early gene), in particular its splice variant IEX-1S engaged in impeding cell division [10,34]. In estrogen-positive breast cancer cells, AMH is also responsible for apoptosis, which was confirmed by increased concentrations of caspase-3 and annexin V [10]. On the other hand, it was found that breast cancer risk increases along with increasing AMH concentration, suggesting this hormone as a possible biomarker for breast cancer [35]. Conflicting results may indicate the involvement of additional mechanisms. It cannot be excluded that the association of high AMH levels with breast cancer may be due to a higher prevalence of PCOS, and not directly of high AMH [36]. This additionally underlines the necessity of AMH and AMHRII investigation in aspects of various patient characteristics, including comorbidities.

Because cancers are genetically and histologically heterogenous, and based on their different growth patterns, various classifications are outlined to help determine prognoses and propose individualized treatments for particular patients [37]. Continuously used for the past 35 years, Bokhman’s classification divides carcinomas into the two types: hormone-dependent (endometrioid and mucous) and hormone-independent, which include poorly prognosticating clear cell, serous, and mixed carcinomas, as well as other rare forms of cancer [38].

Expression of AMHRII elevates from healthy endometrium through endometrial hyperplasia to the highest concentration in endometrial cancer [15]. We did not observe any differences in AMHRII expression depending on the histological type of EC. The presence of AMHRII was confirmed in all cases of PCS (12/12) and EC (218/218), including type 2 EC according to Bokhman’s classification (17/17). Having analyzed the available literature of the subject, one can state that so far, there has been no information on the presence of AMHRII in EC from the following groups: serous (e.g., cell lines: ARK1, ARK2, HEC-155/180, SPEC-2), clear cell, or mixed carcinomas [32]. The studies that have been published confirmed the presence of AMHRII in EC of endometrioid type in 58–75% [17,32]. Despite the histopathological diversity of our samples, the ultimate result pointing to AMHRII expression in all patients may be attributed to the homogenous ethnic background of the patients (Caucasian race) or the chosen methodology, which consisted of applying the technique of tissue microarrays (TMA). The TMAs technique makes it possible to conduct IHC reactions in entirely homogenous standardized conditions.

Concurrent hypertension did not affect AMHRII expression in EC cells. Diabetes mellitus (type 2) was related to lower expression of the receptors. To our knowledge, there are no published studies that compare AMHRII expression in diabetic female patients and no molecular explanation of this observation was proposed. There are, however, studies presenting lower concentrations of serum AMH in type 1 diabetic female patients [39]. Lower concentrations were also reported in PCOS patients with diabetes, in comparison with PCOS patients without diabetes [40]. Attempts at describing this correlation suggested that vascular damage lowers the ovarian reserve [39]. Another study conducted in a group of premenopausal women with diabetes did not prove that markers of early vascular damage correlated with the level of AMH [41]. Further studies are required to determine the exact reasons of decreased AMHRII expression in EC tissue due to type 2 diabetes.

Additionally, it would be a valuable finding to determine a correlation between AMHRII expression and the presence of diabetes in different histological types of cancers and a 5-year survival rate of patients suffering from these cancers. Adding to the increasingly better recognized genetic background of cancers, this differentiation might, if only partly, account for another biological mechanism of some cancers than the one expected, which results from histopathologic type according to Bokhman. The modern taxonomy according to the Cancer Genome Atlas Research, which takes into account the frequency of mutations in particular genes and the number of copies of abnormal protein, classifies carcinomas into four groups: POLE ultramutated, microsatelite insistability hypermutated, copy-number low endometrioid, and copy-number high serous-like [42]. The new taxonomy carries high costs of genetic testing, and has not spread around the world as of yet. The future will show whether AMHRII labeling in EC tissues may become a routine and perhaps valuable procedure, increasing at the same time the chance of implementing a theranostic approach to the patient.

## 5. Conclusions

The anti-Mullerian hormone is a known anticancer agent. It was tested alone or together with classical chemotherapeutics drugs in laboratory settings and it showed improved outcomes of treatment in every type of cancer presenting the receptors for AMH. The efficiency of potential AMH anticancer therapy would be dependent on the expression of its specific AMHRII receptor in the target tissue. Our results confirm AMHRII presence in all histopathological types of EC at all stages of disease and indicate that only diabetes type 2 decreases the concentration of AMHRII in EC tissue.

## Figures and Tables

**Table 1:** Summary of the histopathological type of endometrial lesion, number of patients in each group, and mean ± SD, 95% confident limits of mean, quartiles Q1, Q2, Q3, minimal and maximal values of AMHRII expression.

Histopathological Type of Endometrial Lesion		Patients Number	Mean ± SD	95% C.l.	Q1–Q2–Q3	Min–Max
precancerous state (PCS)	Nonatypical endometrial hyperplasia (NH)	8	5.33 ± 1.782	3.84–6.82	4.0–4.33–7.0	4.00–8.00
	Atypical hyperplasia (AH)	4	6.33 ± 2.000	3.15–9.52	4.67–6.67–8.0	4.00–8.00
type 1 according Bokhman’s	Endometrioid adenocarcinoma G1 (G1)	49	5.69 ± 1.645	5.21–6.16	4.0–5.33–7.33	2.67–8.00
	Endometrioid adenocarcinoma G2 (G2)	146	6.32 ± 1.801	6.03–6.62	4.67–6.67–8.0	2.33–12.00
	Endometrioid adenocarcinoma G3 (G3)	6	6.44 ± 1.559	4.81–8.08	4.67–6.67–8.0	4.67–8.00
type 2 according Bokhman’s	Serous adenocarcinoma (SA)	8	6.25 ± 1.591	4.92–7.58	4.0–4.33–7.0	4.00–8.00
	Clear cell adenocarcinoma (CCA)	4	6.83 ± 1.575	4.33–9.34	5.67–7.33–8.0	4.67–8.00
	Mixed adenocarcinoma (MA)	5	7.60 ± 0.894	6.49–8.71	8.0–8.0–8.0	6.00–8.00

## Supplementary Materials

The following are available online at https://www.mdpi.com/2073-4409/9/10/2312/s1, Table S1. Demographic traits of woman distinguished according to the histopathological type of the cancer; in the Table shows: mean± SD, median (Q2), minimum and maximum values, N—sample size. Table S2. Demographic traits of woman distinguished according to the FIGO classification; in the Table shows: mean ± SD, median (Q2), minimum and maximum values, N—sample size. Table S3. Demographic traits of woman distinguished according to occurrence of diabetes type 2; in the Table shows: mean± SD, median (Q2), minimum and maximum values, N—sample size. Figure S1. Distribution of AMHRII expression level in groups of patients distinguished according to histopathological type of endometrial lesion; above the figure shows the results of testing the comparison of the distribution with the normal distribution using the Shapiro-Wilk test. Figure S2. Distribution of AMHRII expression level in groups of patients distinguished according to FIGO classification; above the figure shows the results of testing the comparison of the distribution with the normal distribution using the Shapiro-Wilk test.
