# Supplementary material for: Anti-Müllerian Hormone Type II Receptor Expression in Endometrial Cancer Tissue
Source: Cells. 2020 Oct 17;9(10):2312. doi: 10.3390/cells9102312 (PMC7603004; doi:10.3390/cells9102312)
Supplement: Supplementary file 1 [file cells-09-02312-s001.zip › s/Supplementary Tables S1.pdf]

Table S1. Demographic traits of woman distinguished according to the histopathological type of the cancer; in the Table shows: mean± SD, median (Q2), minimum and maximum values, N – sample size

| Type histopathological of cancer | N | Age (years) | BMI (mass / height$^2$) | Years of menstruation (years) | Number of births (n) | Mass of newborn (g) | Average time of breastfeeding (months) | Total time of breastfeeding (months) |
|---|---|---|---|---|---|---|---|---|
| NH | 8 | 59.0±9.71;<br>56.5;<br>47-75 | 29.78±3.963;<br>30.1;<br>23.4-34.7 | 36.1±3.83;<br>36.0;<br>32-43 | 2.7±1.58;<br>2;<br>1-6 | 3367.5±480.56;<br>3450;<br>2350-3950 | 4.4±2.82;<br>3.5;<br>2-11 | 13.1±13.27;<br>9.0;<br>3-44 |
| AH | 4 | 53.0±3.65;<br>53.0;<br>49-57 | 24.58±1.357;<br>24.6;<br>23.0-26.2 | 37.8±4.57;<br>37.5;<br>33-43 | 2.0±0.82;<br>2;<br>1-3 | 3455.0±373.85;<br>3485;<br>3000-3850 | 3.8±1.50;<br>3.0;<br>3-6 | 7.5±3.87;<br>7.5;<br>3-12 |
| G1 | 49 | 60.3±9.39;<br>59.0;<br>42-83 | 30.81±5.825;<br>31.1;<br>20.0-45.9 | 35.8±4.75;<br>36.0;<br>14-44 | 2.7±1.68;<br>2;<br>0-7 | 3510.0±426.49;<br>3510;<br>2700-4790<br>*N=42 | 7.2±6.14;<br>6.0;<br>0-21<br>*N=47 | 23.9±23.75;<br>13.0;<br>0-80<br>*N=47 |
| G2 | 146 | 63.0±9.87;<br>63.0;<br>35-87 | 30.74±5.837;<br>30.2;<br>18.3-49.2 | 36.6±5.16;<br>37.0;<br>17-49 | 2.5±1.57;<br>2;<br>0-8 | 3431.2±486.53;<br>3500;<br>1900-4760<br>*N=129 | 5.9±7.24;<br>3.0;<br>0-48<br>*N=145 | 18.5±26.70;<br>6.0;<br>0-144<br>*N=145 |
| G3 | 6 | 60.5±8.02;<br>60.5;<br>48-72 | 29.08±6.385;<br>27.4;<br>21.3-39.0 | 37.5±4.89;<br>37.5;<br>30-43 | 2.8±1.33;<br>3;<br>1-5 | 3463.3±677.54;<br>3715;<br>2600-4100 | 7.7±8.59;<br>3.0;<br>1.5-22.0 | 23.3±26.10;<br>12.4;<br>2-66 |
| SA | 8 | 69.6±8.52;<br>71.5;<br>56-80 | 30.05±6.582;<br>28.4;<br>21.6-40.4 | 36.6±3.29;<br>36.5;<br>30-41 | 4.8±1.49;<br>4.5;<br>3-7 | 3553.8±453.49;<br>3595;<br>2960-4140 | 6.4±4.69;<br>6.0;<br>1.5-12.0 | 29.7±25.93;<br>23.4;<br>6-84 |
| CCA | 4 | 71.2±5.38;<br>71.2;<br>65-78 | 25.48±6.956;<br>25.4;<br>17.1-34.1 | 36.2±2.22;<br>36.0;<br>34-39 | 2.5±1.00;<br>2;<br>2-4 | 3482.5±363.17;<br>3525;<br>3000-3880 | 10.3±5.50;<br>8.6;<br>6-18 | 24.0±10.08;<br>23.9;<br>12-36 |
| MA | 5 | 63.0±7.78; | 31.76±4.396; | 39.8±5.89; | 4.2±4.38; | 3620.0±480.62; | 6.0±4.64; | 24.4±28.05; |

| | | | | | | | |
|---|---|---|---|---|---|---|---|
| | 65.0;<br>51-72 | 32.9;<br>26.1-37.1 | 37.0;<br>34-49 | 2;<br>2-12 | 3530;<br>3040-4150 | 6.0;<br>1-12 | 18.0;<br>2-72 |
