# Supplementary material for: Anti-Müllerian Hormone Type II Receptor Expression in Endometrial Cancer Tissue
Source: Cells. 2020 Oct 17;9(10):2312. doi: 10.3390/cells9102312 (PMC7603004; doi:10.3390/cells9102312)
Supplement: Supplementary file 1 [file cells-09-02312-s001.zip › s/Supplementary Tables S2.pdf]

Table S2. Demographic traits of woman distinguished according to the FIGO classification; in the Table shows: mean± SD, median (Q2), minimum and maximum values, N – sample size

FIGO	N	Age (years)	BMI (mass / height^2)	Years of menstruation (years)	Number of births (n)	Mass of newborn (g)	Average time of breastfeeding (months)	Total time of breastfeeding (months)
0	14	57.3±7.95; 55.5; 47-75	30.13±6.207; 30.0; 23.0-34.7	36.6±3.69; 36.0; 32-43	0.1±0.36; 0; 0-1	3343.6±438.91; 3400; 2350-3950	4.7±2.58; 3.5; 2-11	11.8±10.39; 9.0; 3-44
IA	99	60.8±9.38; 59.0; 39-83	30.95±5.879; 30.4; 17.1-49.2	36.6±4.52; 37.0; 22-44	0.3±0.68; 0 0-3	3453.0±450.30; 3500; 2300-4760 *N=89	6.3±6.15; 4.5; 0-24 *N=97	20.9±25.93; 12.0; 0-144 *N=97
IB	57	63.8±8.99; 65.0; 42-83	30.20±5.586; 29.7; 20.9-45.9	35.9±4.96; 37.0; 17-47	0.3±0.60; 0; 0-2	3494.5±518.98; 3500; 1900-4790 *N=49	6.1±6.75; 3.0; 0-24 *N=56	19.2±25.60; 8.0; 0-91.8 *N=56
II	29	67.0±9.01; 67.0; 51-87	31.82±6.702; 31.2; 18.3-48.3	37.2±5.78; 38.0; 14-47	0.2±0.49; 0; 0-2	3391.6±524.71; 3300; 1980-4250 *N=25	3.9±5.29; 1.5; 0-24 *N=29	15.1±25.50; 5.0; 0-120 *N=29
IIIA	9	59.7±14.76; 58.0; 35-80	27.83±6.023; 28.6; 19.5-37.1	33.1±6.17; 36.0; 20-38	0.2±0.44; 0; 0-1	3716.2±323.37; 3625; 3310-4250 *N=8	6.6±5.77; 6.0; 0-18	20.4±26.25; 12.0; 0-84
IIIB	3	64.0±14.00; 70.0; 48-74	32.27±9.860; 35.1; 21.3-40.4	36.7±1.53; 36.0; 34-37	0; 0; 0-0	3416.7±693.42; 3600; 2650-4000	9.7±8.02; 9.0; 2-18	39.7±35.30; 45.0; 2-72
IIIC1	14	65.9±8.89; 65.5; 51-81	30.02±4.196; 29.0; 24.8-37.8	39.4±5.96; 40.5; 28-49	0.3±0.61; 0; 0-2	3383.1±458.77; 3200; 2800-4100	8.8±7.33; 9.4; 0-24	30.0±28.10; 30.5; 0-96

						*N=13		
IIIC2	2	65.0±14.14;<br>65.0;<br>55-75	24.95±1.626;<br>25.0;<br>23.8-26.1	36.5±0.71;<br>36.5;<br>36-37	0;<br>0;<br>0-0	3470.0±268.70;<br>3470;<br>3280-3660	5.6±7.99;<br>5.6;<br>0-11.3	17.0±23.97;<br>17.0;<br>0-33.9
IVB	3	60.0±14.73;<br>52.0;<br>51-77	30.13±6.207;<br>29.8;<br>24.1-36.5	36.0±3.61;<br>37.0;<br>32-39	0;<br>0;<br>0-0	3666.7±152.75;<br>3700;<br>3500-3800	19.0±25.16;<br>6.0;<br>3-48	23.0±22.91;<br>18.0;<br>3-48
