# Supplementary material for: Anti-Müllerian Hormone Type II Receptor Expression in Endometrial Cancer Tissue
Source: Cells. 2020 Oct 17;9(10):2312. doi: 10.3390/cells9102312 (PMC7603004; doi:10.3390/cells9102312)
Supplement: Supplementary file 1 [file cells-09-02312-s001.zip › s/Supplementary Tables S3.pdf]

Table S3. Demographic traits of woman distinguished according to occurrence of diaabetes type 2; in the Table shows: mean± SD, median (Q2), minimum and maximum values, N – sample size

Diabetes type 2	N	Age (years)	BMI (mass / height^2)	Years of menstruation (years)	Number of births (n)	Mass of newborn (g)	Average time of breastfeeding (months)	Total time of breastfeeding (months)
No	180	61.3±9.86;<br>60.0;<br>35-87	29.58±5.644;<br>29.2;<br>17.1-49.2	36.5±5.06;<br>37.0;<br>14-49	2.5±1.60;<br>2;<br>0-8	3419.7±477.27;<br>3500;<br>1900-4790<br>*N=161	5.9±6.59;<br>3.0;<br>0-48<br>*N=179	17.8±22.91;<br>9.0;<br>0-144<br>*N=179
Yes	50	66.5±8.10;<br>66.0;<br>51-83	33.69±5.211;<br>33.2;<br>25.0-48.3	36.6±4.47;<br>37.0;<br>28-49	3.1±2.00;<br>3;<br>0-12	3588.4±423.14;<br>3630;<br>2830-4760<br>*N=45	7.4±7.17;<br>5.5;<br>0-24<br>*N=48	28.2±31.61;<br>18.0;<br>0-120<br>*N=48
