# Supplementary figures and images for: Anti-Müllerian Hormone Type II Receptor Expression in Endometrial Cancer Tissue
Source: Cells. 2020 Oct 17;9(10):2312. doi: 10.3390/cells9102312 (PMC7603004; doi:10.3390/cells9102312)

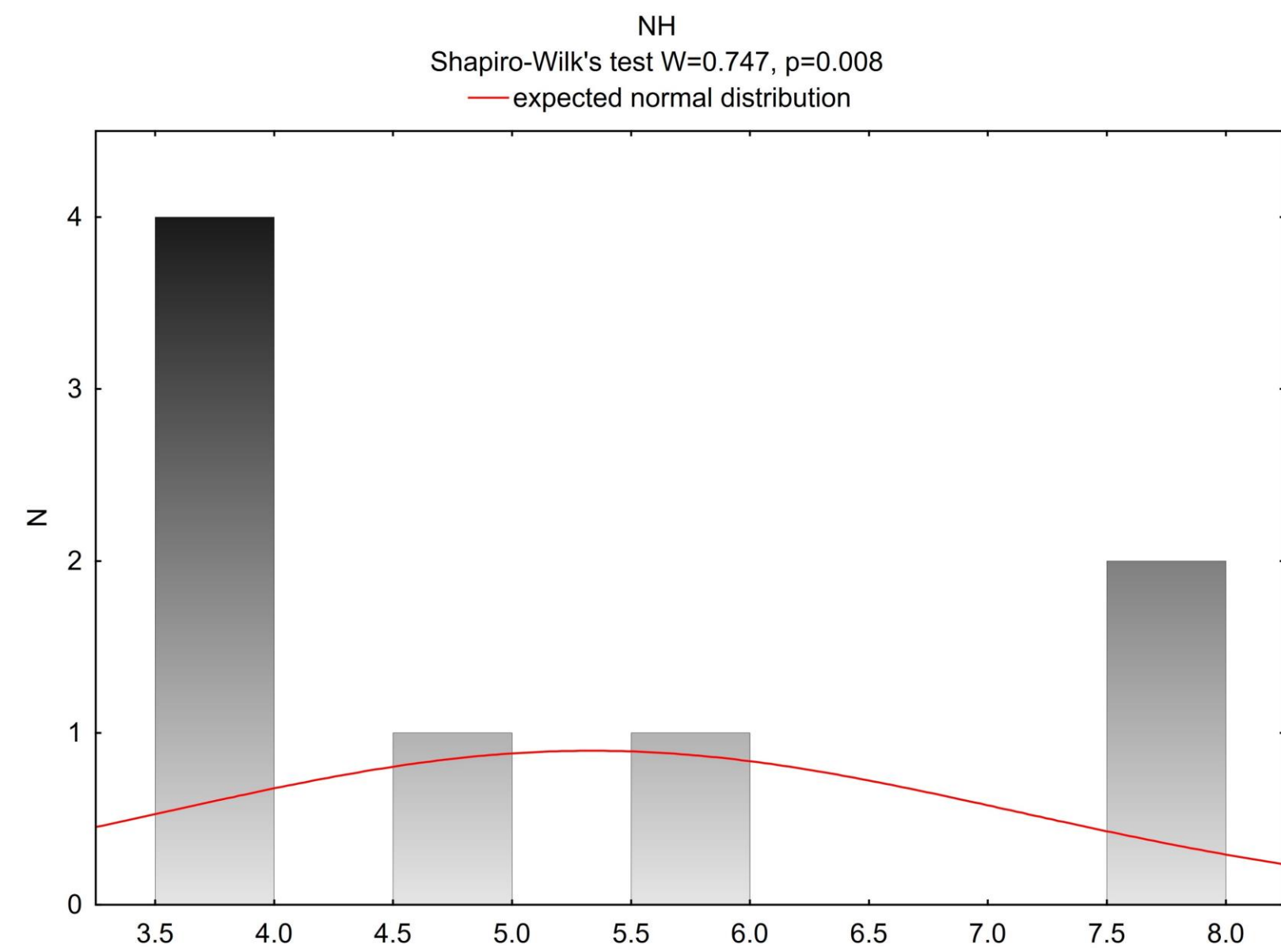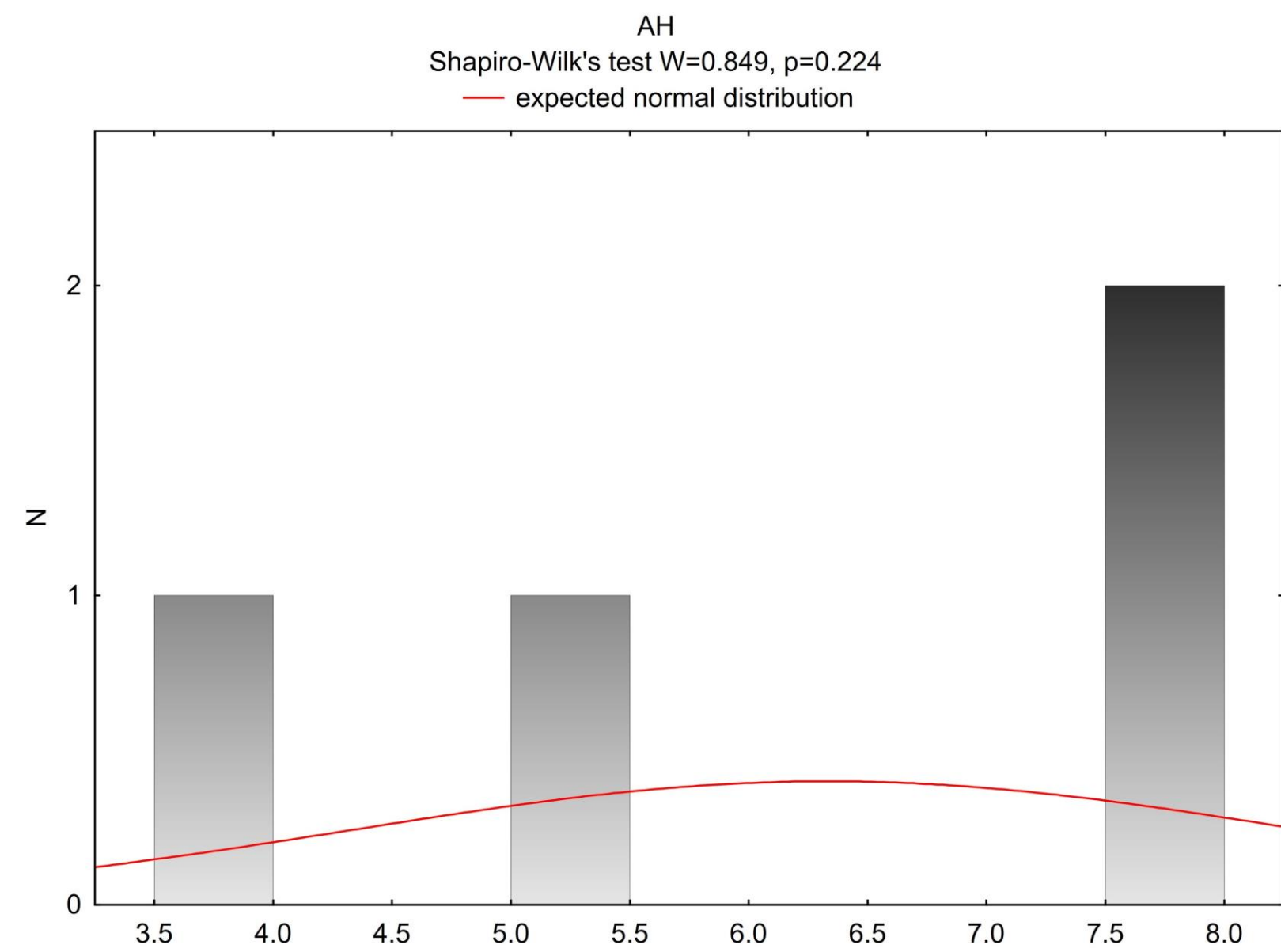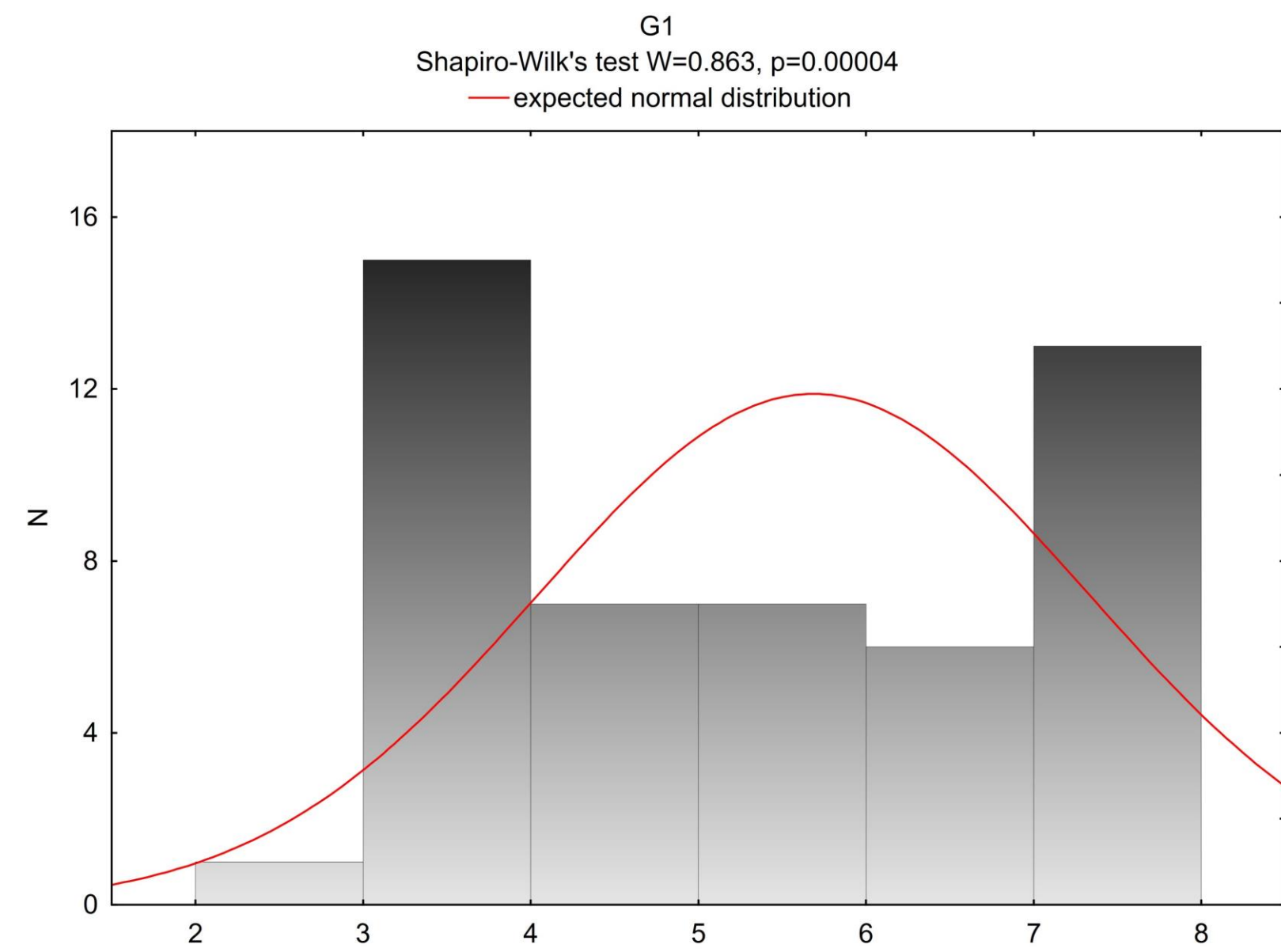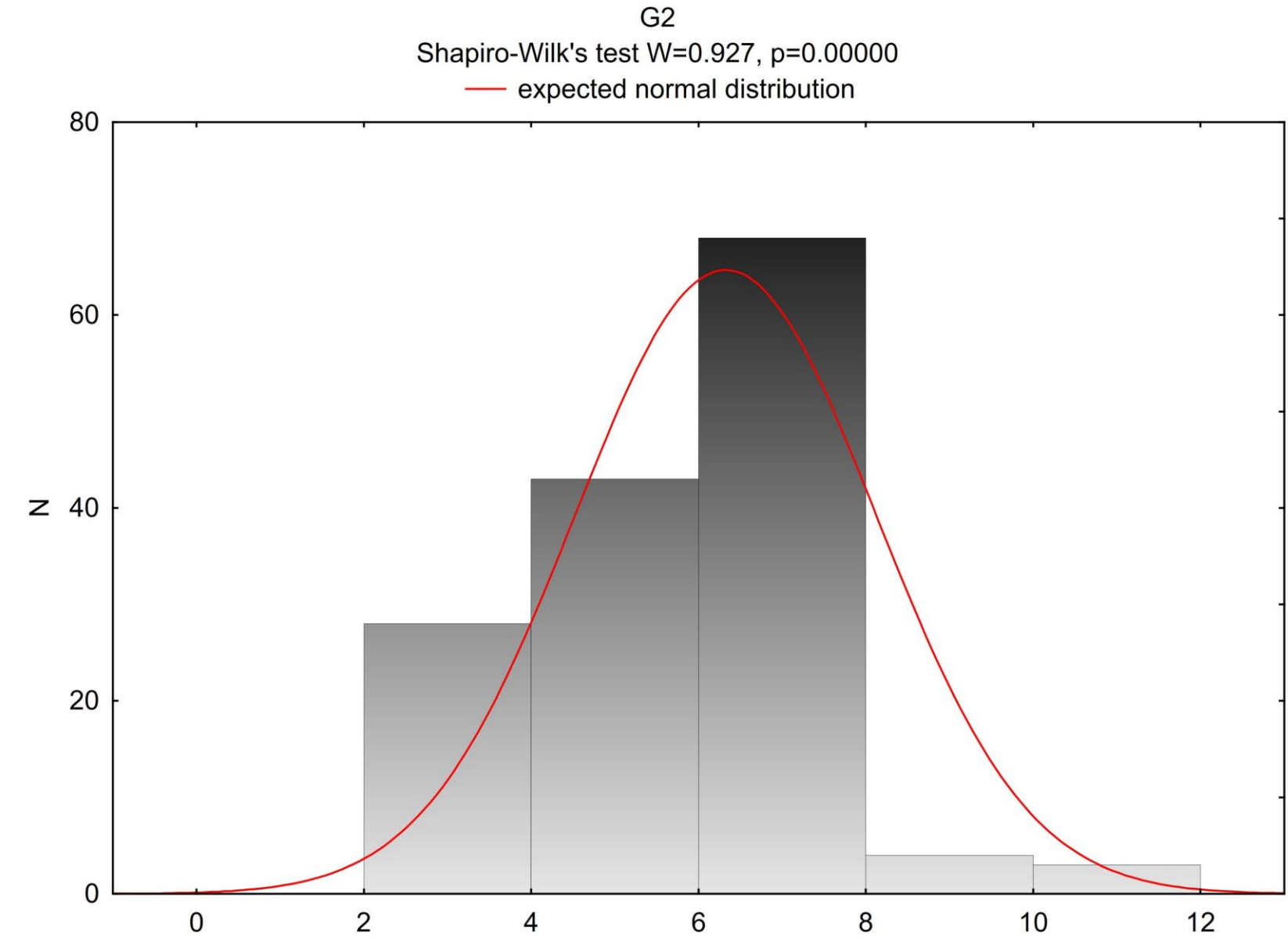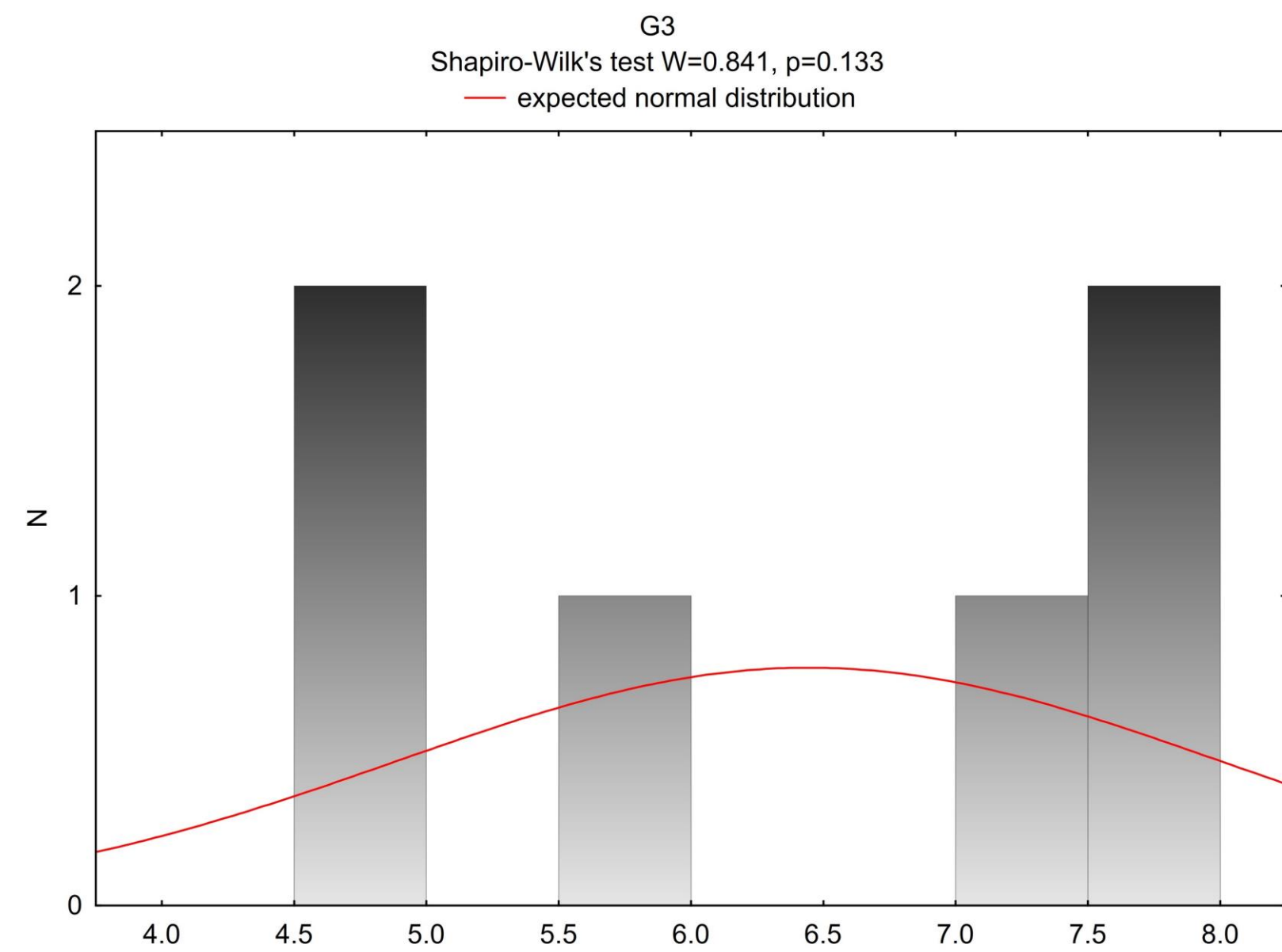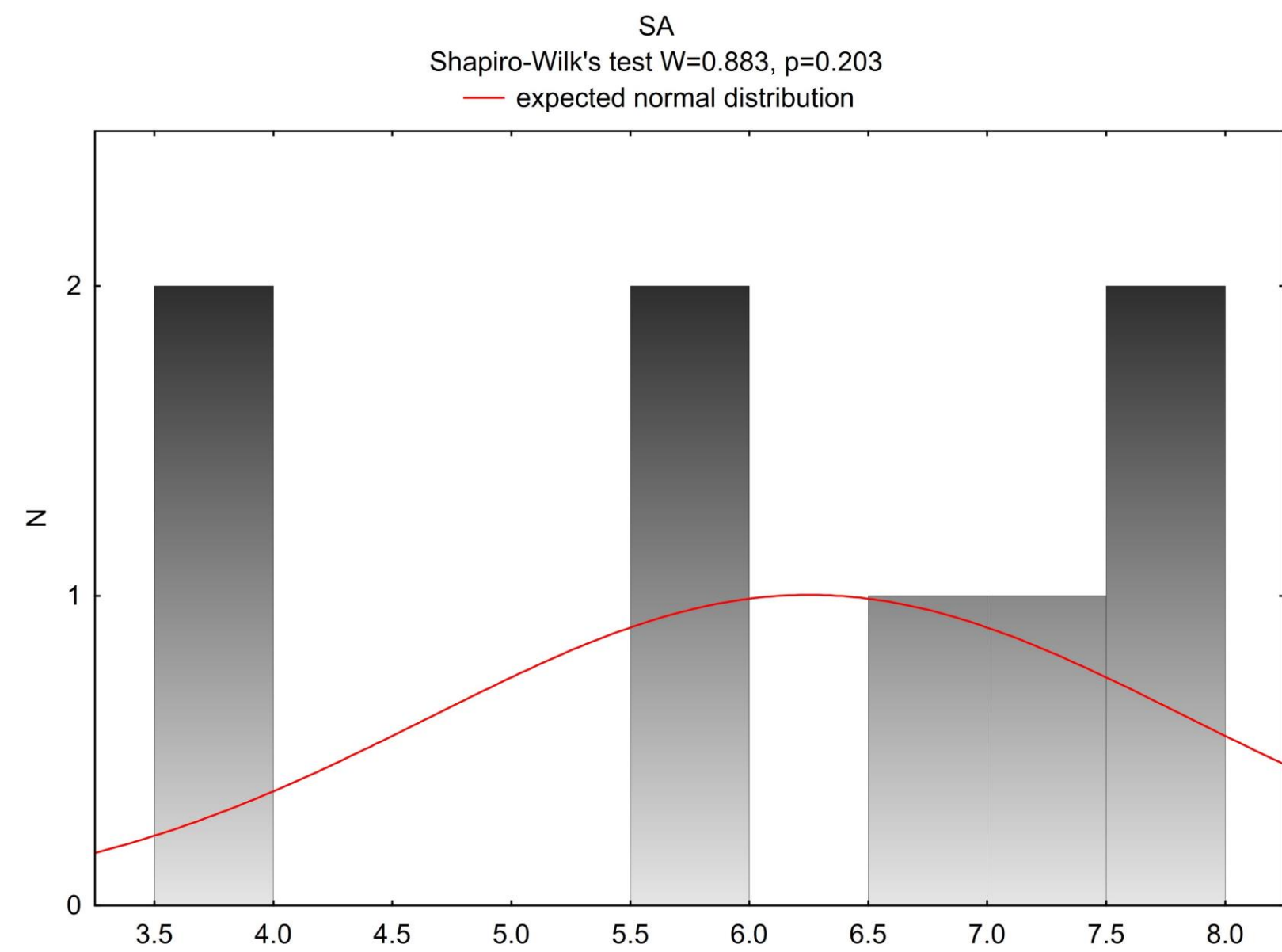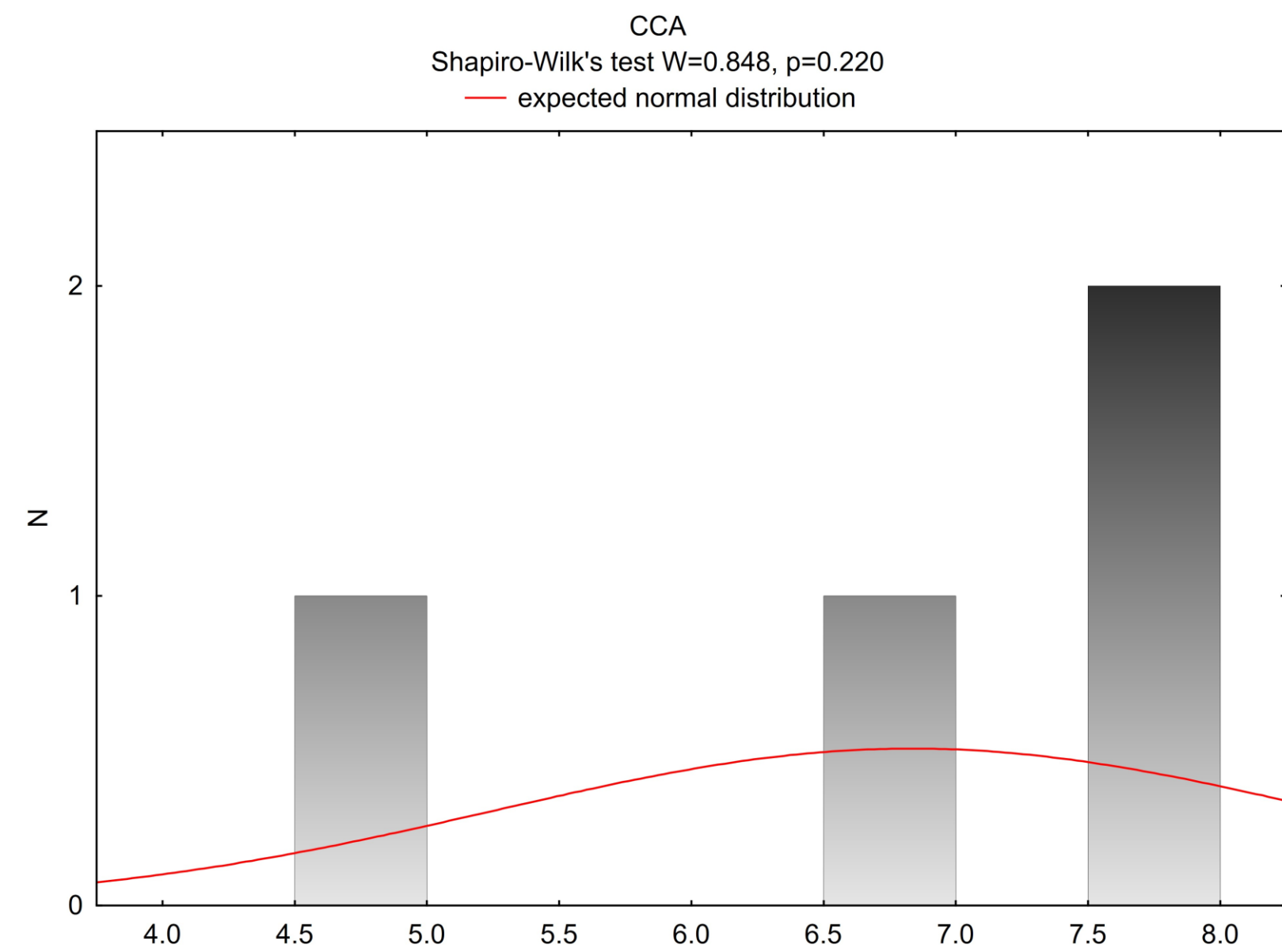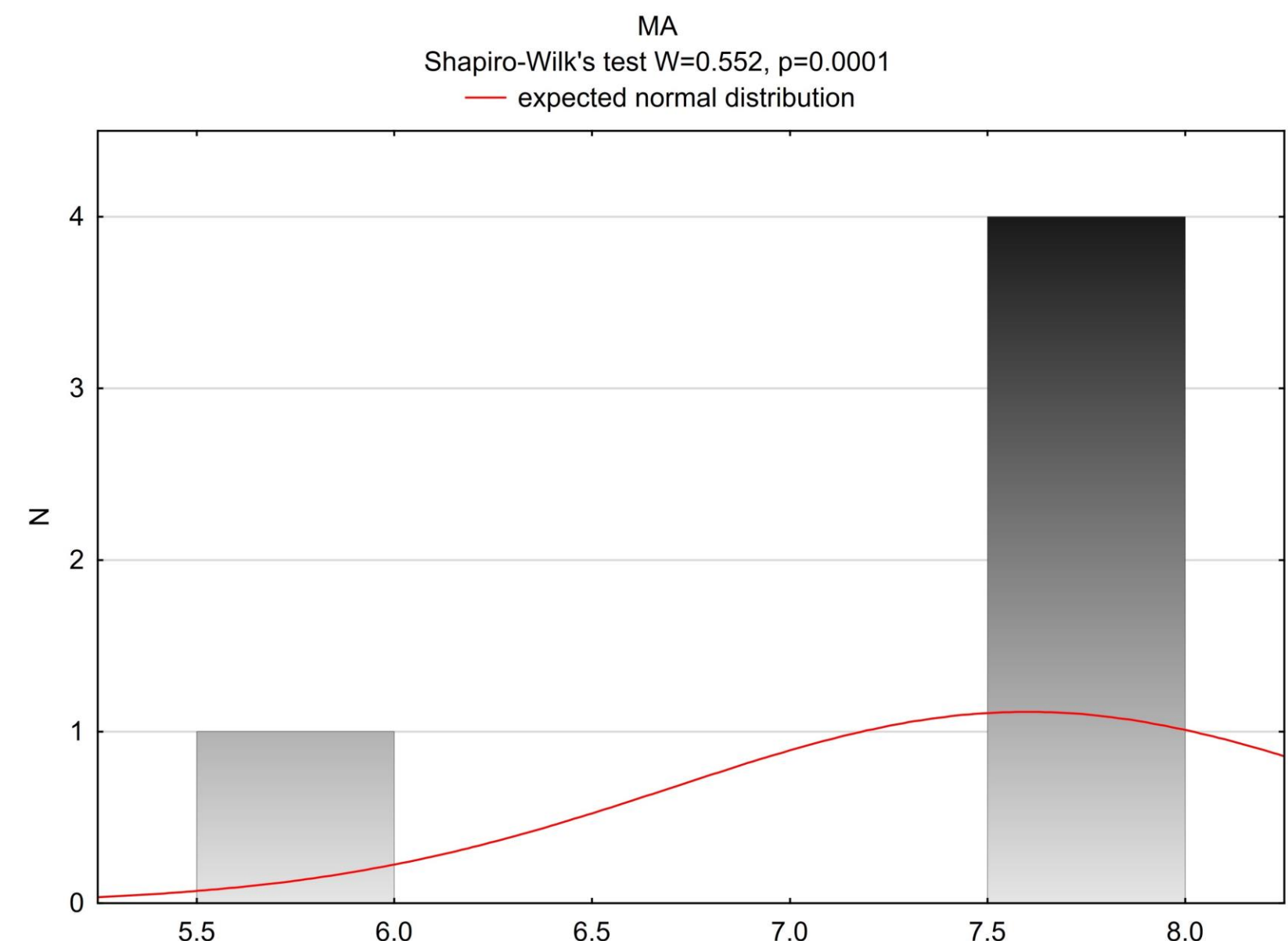

Supplement: Supplementary file 1 [file cells-09-02312-s001.zip › s/Supplementary Figure 1.pdf]

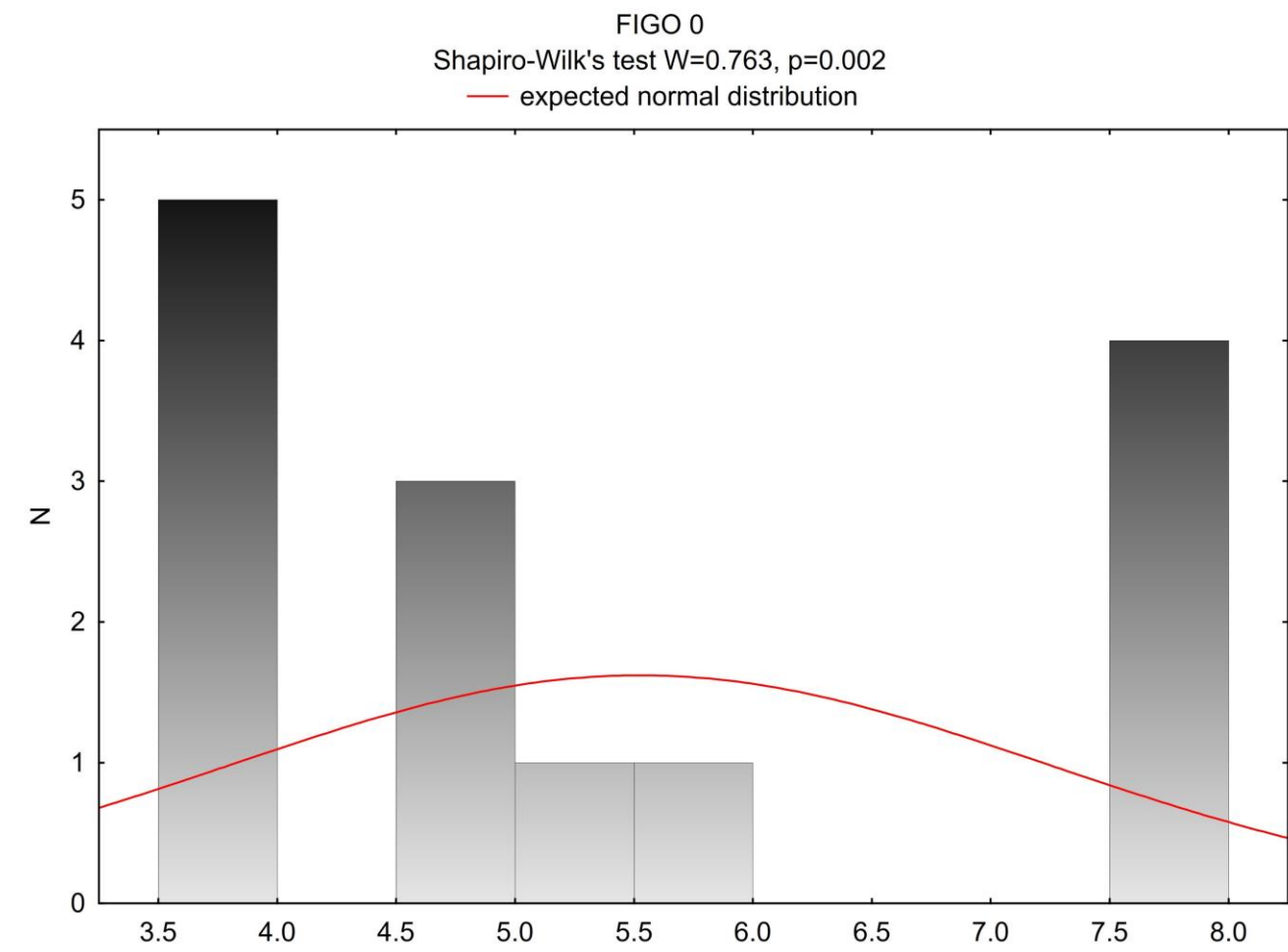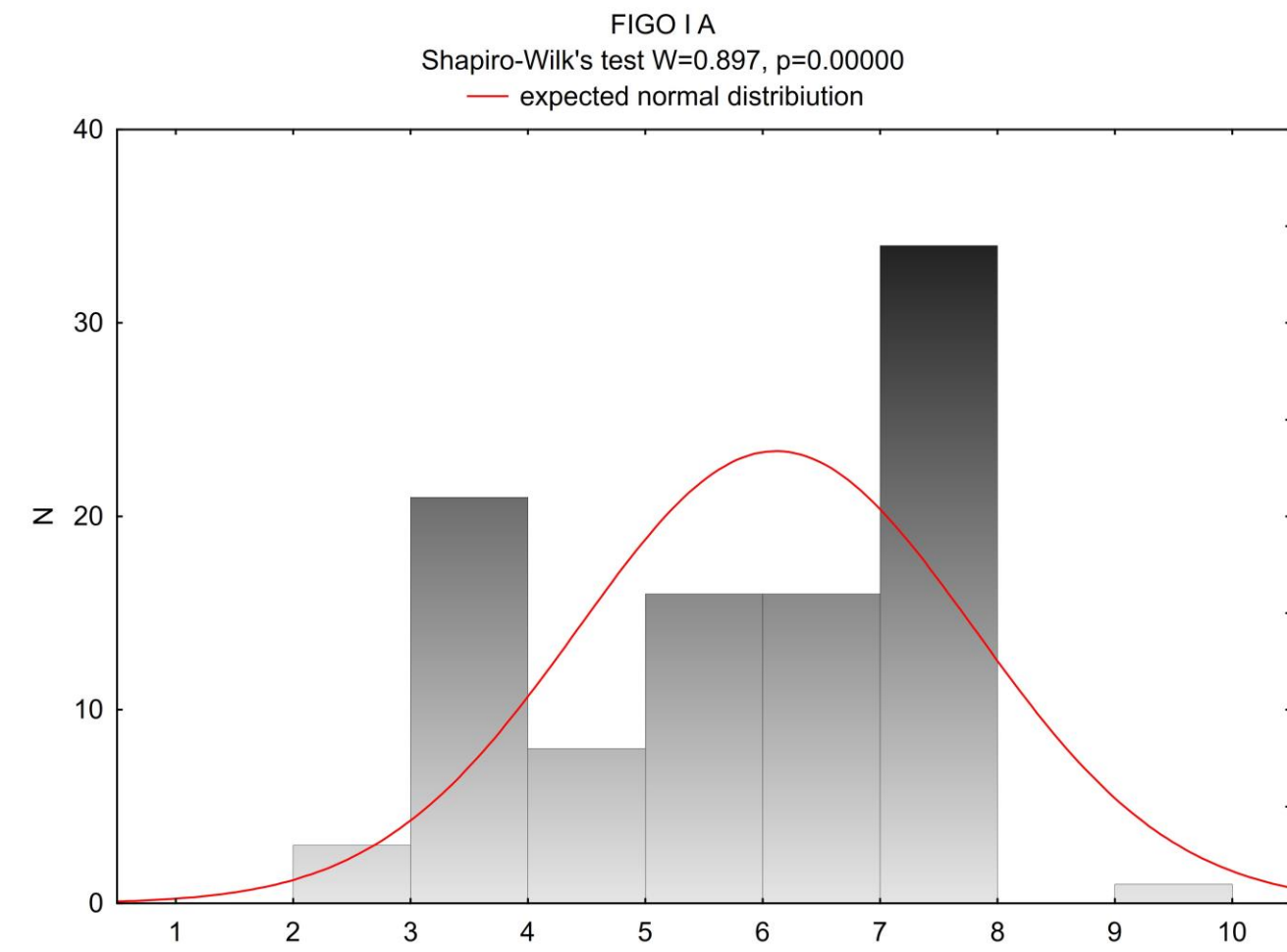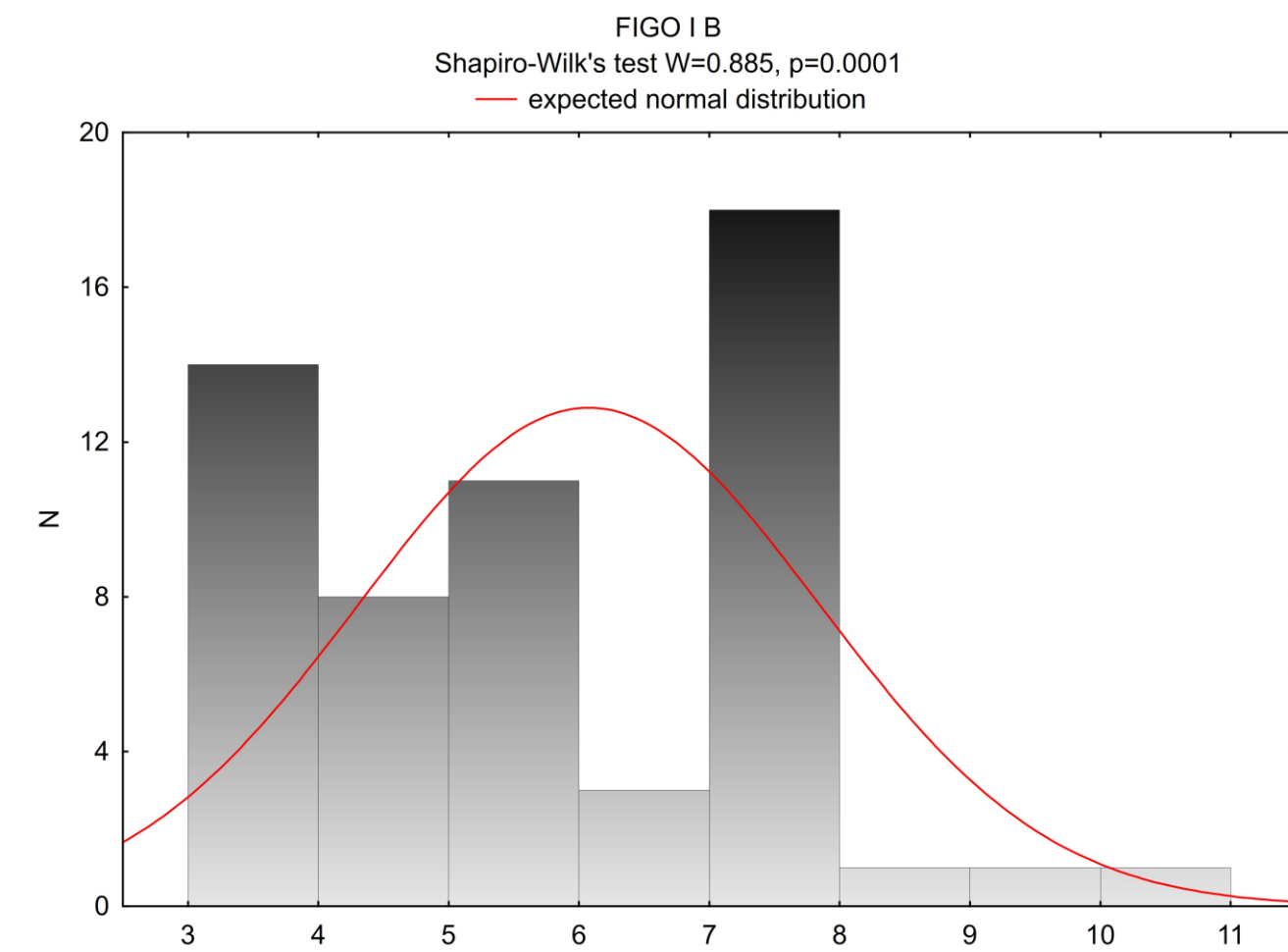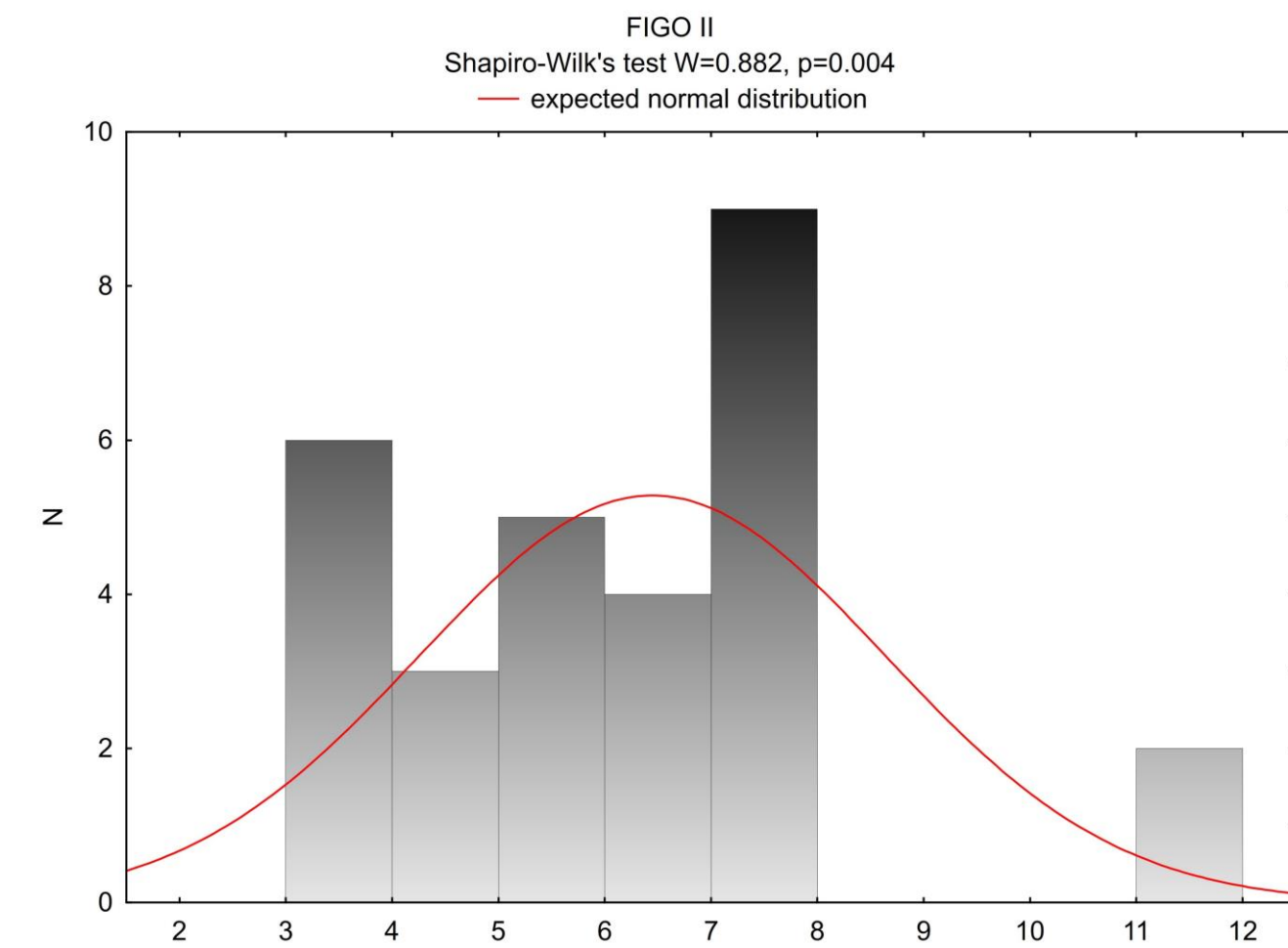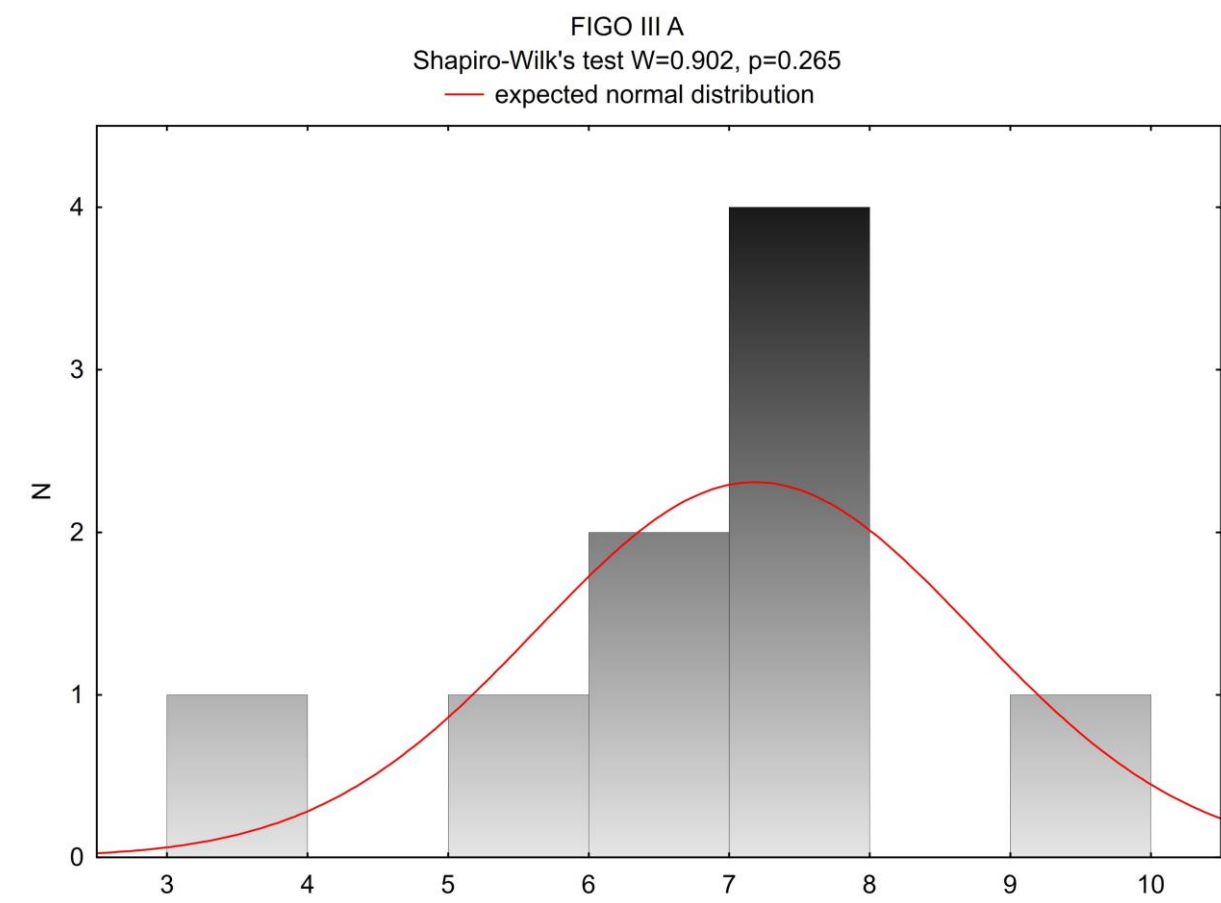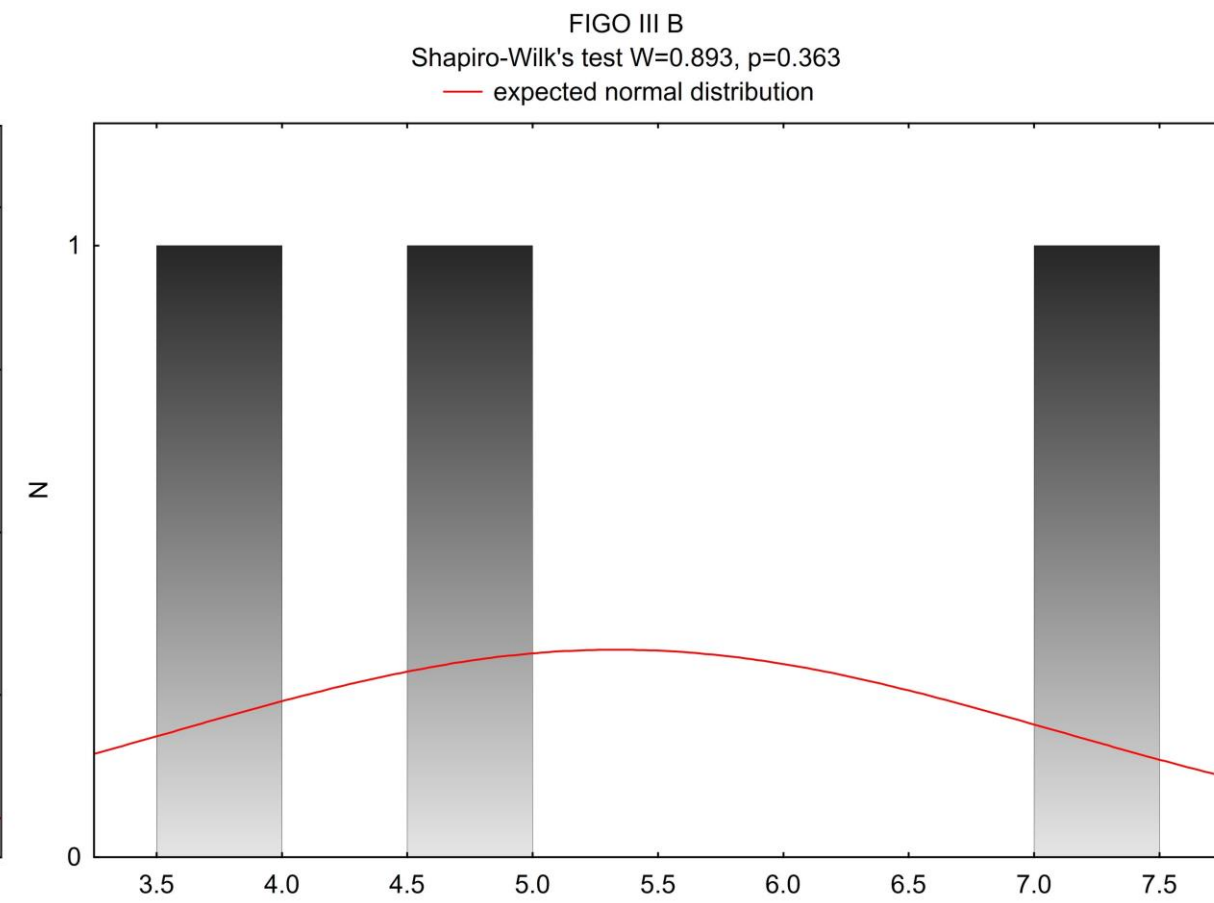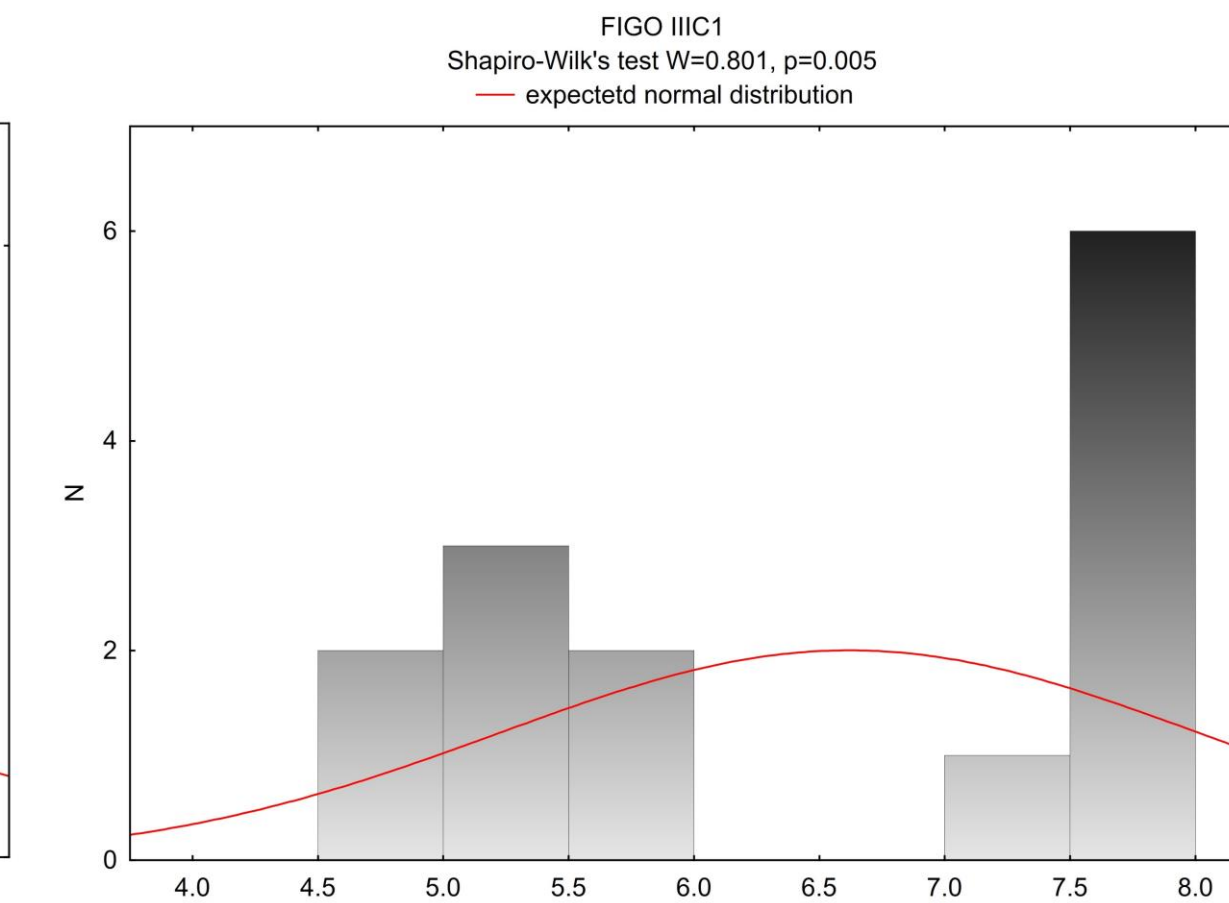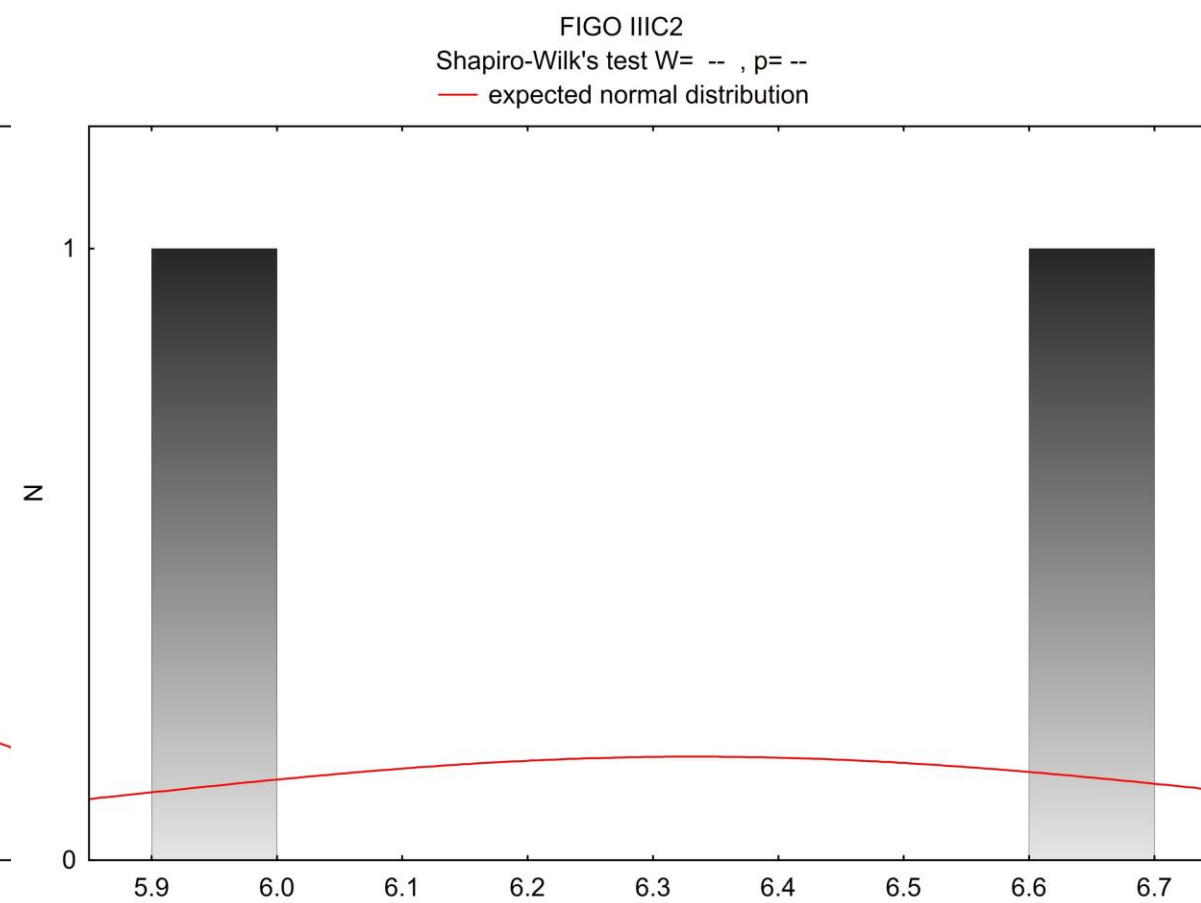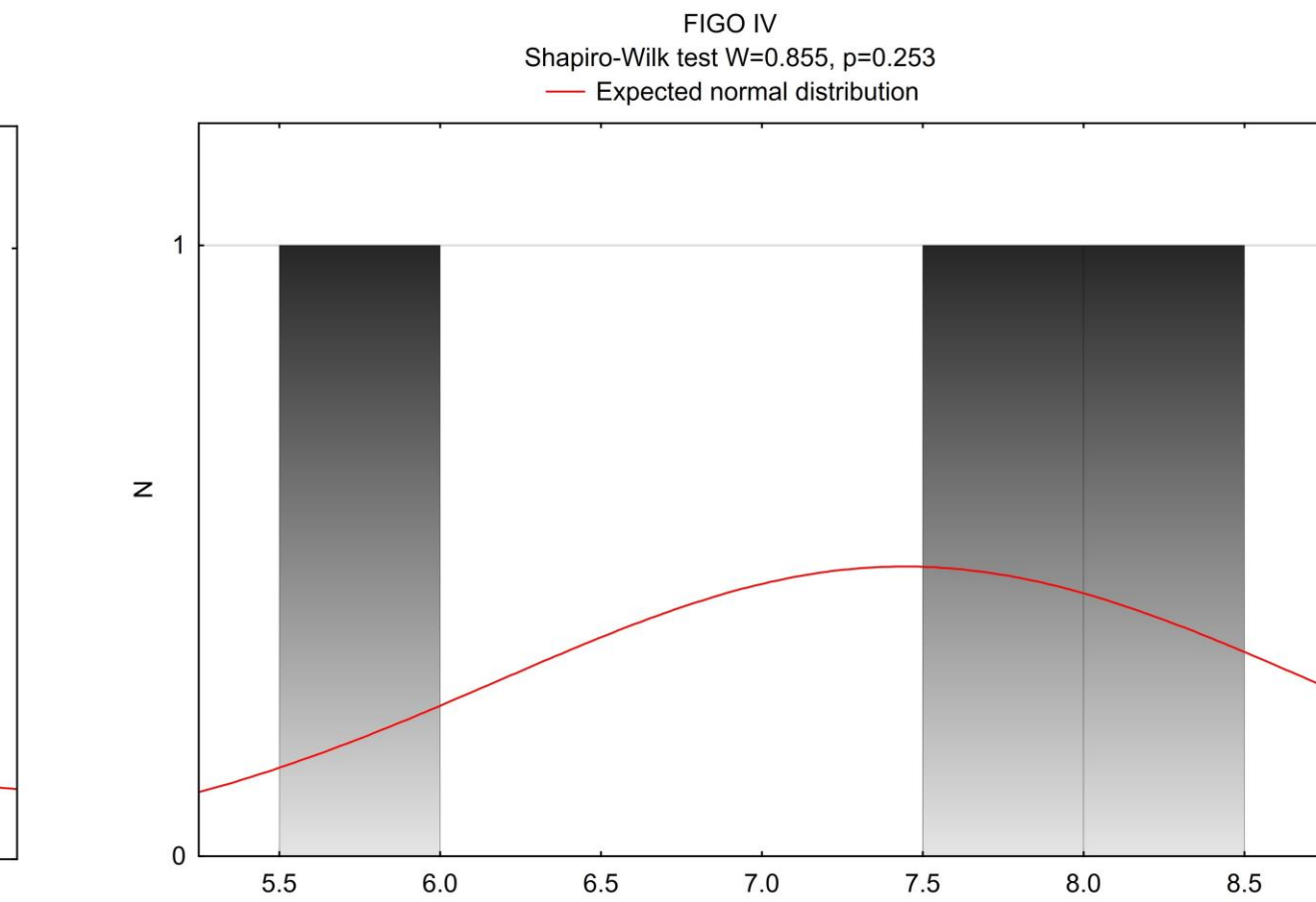

Supplement: Supplementary file 1 [file cells-09-02312-s001.zip › s/Supplementary Figure 2.pdf]
